# Identification of Attenuators of Transcriptional Termination: Implications for RNA Regulation in Escherichia coli

**DOI:** 10.1128/mbio.02371-22

**Published:** 2022-10-13

**Authors:** Teppei Morita, Nadim Majdalani, Masahiro C. Miura, Rerina Inose, Taku Oshima, Masaru Tomita, Akio Kanai, Susan Gottesman

**Affiliations:** a Institute for Advanced Biosciences, Keio University, Tsuruoka, Yamagata, Japan; b Graduate School of Media and Governance, Keio University, Fujisawa, Kanagawa, Japan; c Laboratory of Molecular Biology, Center for Cancer Research, National Cancer Institute, Bethesda, Maryland, USA; d Department of Biotechnology, Toyama Prefectural University, Imizu, Toyama, Japan; e Faculty of Environment and Information studies, Keio University, Fujisawa, Kanagawa, Japan; Friedrich-Schiller-Universität Jena; University of Würzburg

**Keywords:** small RNA, transcription termination, SgrS, cold shock protein, Rho

## Abstract

The regulatory function of many bacterial small RNAs (sRNAs) requires the binding of the RNA chaperone Hfq to the 3′ portion of the sRNA intrinsic terminator, and therefore sRNA signaling might be regulated by modulating its terminator. Here, using a multicopy screen developed with the terminator of sRNA SgrS, we identified an sRNA gene (*cyaR*) and three protein-coding genes (*cspD*, *ygjH*, and *rof*) that attenuate SgrS termination in Escherichia coli. Analyses of CyaR and YgjH, a putative tRNA binding protein, suggested that the CyaR activity was indirect and the effect of YgjH was moderate. Overproduction of the protein attenuators CspD and Rof resulted in more frequent readthrough at terminators of SgrS and two other sRNAs, and regulation by SgrS of target mRNAs was reduced. The effect of Rof, a known inhibitor of Rho, was mimicked by bicyclomycin or by a *rho* mutant, suggesting an unexpected role for Rho in sRNA termination. CspD, a member of the cold shock protein family, bound both terminated and readthrough transcripts, stabilizing them and attenuating termination. By RNA sequencing analysis of the CspD overexpression strain, we found global effects of CspD on gene expression across some termination sites. We further demonstrated effects of endogenous CspD under slow growth conditions where *cspD* is highly expressed. These findings provided evidence of changes in the efficiency of intrinsic termination, confirming this as an additional layer of the regulation of sRNA signaling.

## INTRODUCTION

The expression of bacterial genes is controlled, allowing rapid adaptation to environmental changes. Adaptation contributes to the success of biological processes, including those necessary for pathogenesis. Although transcription initiation is the principal regulated event in bacteria, studies have established that regulation of gene expression often involves posttranscriptional mechanisms that occur following transcription initiation, such as transcription elongation and termination, translation, and mRNA degradation. Small regulatory RNAs (sRNAs), which range from 50 to 200 nucleotides in size, play important roles as posttranscriptional regulators of gene expression (1–3). This posttranscriptional regulation by most sRNAs is triggered by their base pairing with mRNA targets, resulting in changes in mRNA stability and translation efficiency (4, 5). Base pair formation in Escherichia coli is typically facilitated by the RNA chaperone protein Hfq (6). Hfq also has roles in protecting sRNAs from the attack of ribonucleases and recruiting RNase E to degrade the coupled target mRNAs and sRNA (7, 8).

Many genes encoding an sRNA are expressed from dedicated promoters that are regulated in response to cellular stresses. Regulation of transcriptional initiation is a key step for increasing sRNAs in response to stress. For instance, the transcription factor SgrR is activated in response to accumulation of glucose-phosphate and stimulates the transcription from the *sgrS* promoter to produce SgrS sRNA (9). SgrS negatively regulates mRNAs of glucose metabolism genes, such as *ptsG*, encoding the major glucose transporter, and positively regulates *yigL*, encoding a HAD-like phosphatase (9–11). This regulation by SgrS results in attenuation of glucose-phosphate stress. Some sRNAs are processed from the 3′ end of mRNAs, tying their transcription to that of the upstream mRNA (12, 13). The transcription of sRNA genes is commonly terminated by intrinsic termination (14), regardless of the pathways for the formation of the 5′ end.

Termination of transcription in E. coli happens either by intrinsic (Rho or factor-independent) or Rho-dependent mechanisms (15, 16). An intrinsic terminator consists of an RNA stem-loop structure followed by a U-rich tract in the nascent RNA, which are elements that are responsible for the dissociation of RNA polymerase from both the DNA template and the nascent RNA (17). Most sRNAs have a moderate-strength stem-loop, followed by a stretch of seven or more Us (18). The poly(U) tail of seven or more Us allows efficient binding by Hfq (19). The individual uridines, including the 3′-terminal hydroxyl group of an sRNA poly(U) tail, bind to the proximal face of the Hfq hexamer (20, 21). The importance of the poly(U) tail can be seen in studies of SgrS. While the terminated product of SgrS, which contains a poly(U) tail, binds Hfq, resulting in a functional sRNA, transcriptional readthrough products of SgrS are nonfunctional as sRNAs, since the poly(U) tract is embedded in the transcript (22). For SgrS, the readthrough transcript is thought to function as an mRNA for the downstream *setA* gene, which encodes a putative sugar transporter, SetA (22, 23). Thus, whether a transcript from an sRNA gene functions as an sRNA or an mRNA (or is rapidly degraded) can be determined by the efficiency of intrinsic termination at its 3′ end.

Our studies on the critical role of proper transcription termination for sRNAs led us to consider that sRNA signaling might be regulated by factors that affect intrinsic termination. This assumption is supported by observations that the efficiency of intrinsic termination of sRNAs is modulated by some physiological and stress signals (22, 24). However, genetic factors and mechanisms by which the transcription termination of sRNAs could be regulated are yet to be explored. Here, we carried out a multicopy screen in E. coli to identify factors that attenuate the intrinsic termination of SgrS, resulting in less production of functional SgrS. Several genes were isolated as multicopy attenuators, and four genes (*cspD*, *ygjH*, *rof*, and *cyaR*) were further studied. We show the effects of each factor on both intrinsic termination of three sRNAs including SgrS and the regulation by SgrS of the target mRNAs. We also demonstrate that one attenuator, CspD, has global effects, promoting transcription extension across some termination sites within operons or internal to genes. Our findings suggest novel regulatory mechanisms of transcriptional termination and additional levels of modulation of sRNA action.

## RESULTS

### Method to screen for genes that affect intrinsic termination of SgrS.

**FIG 1:**
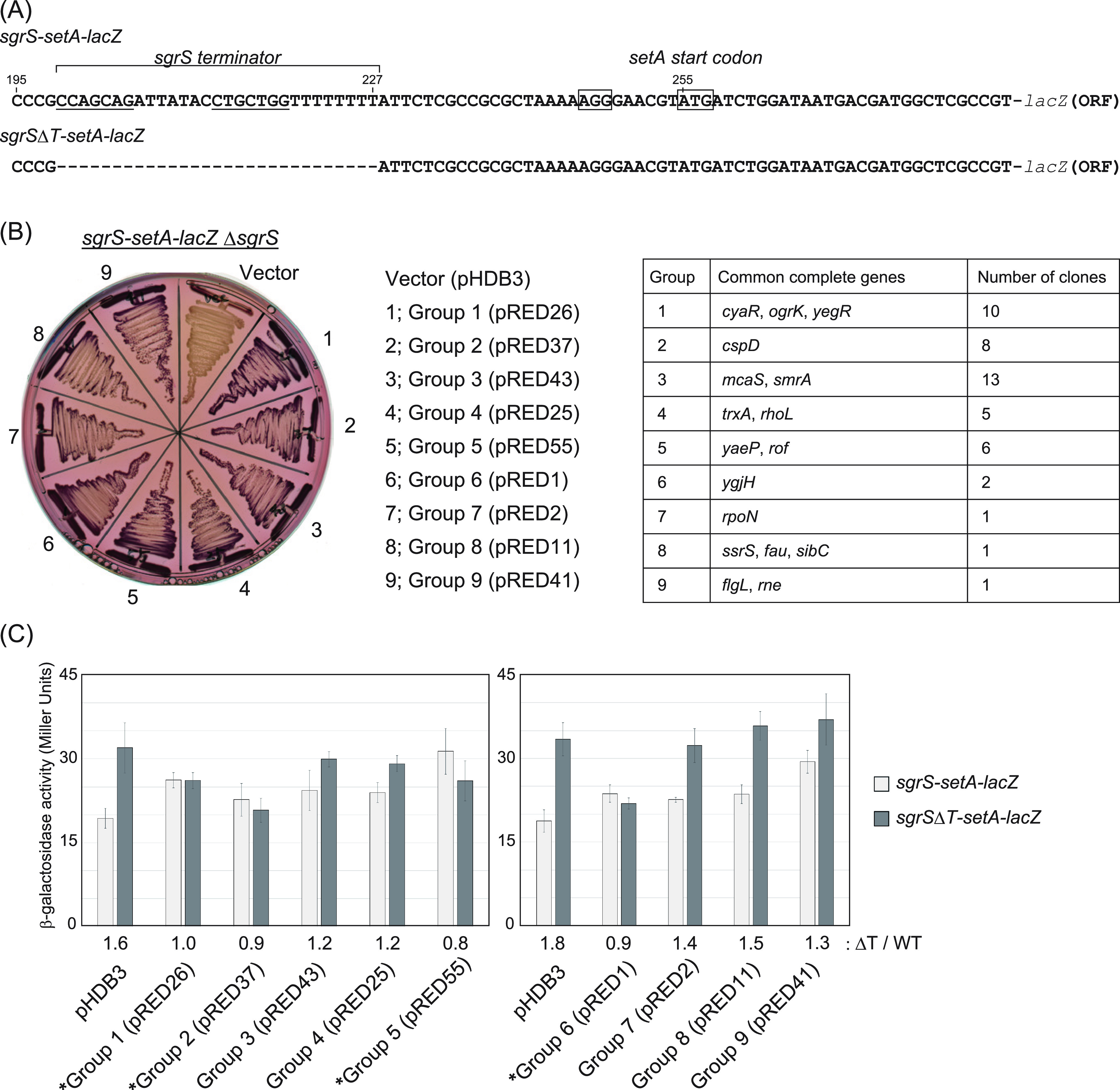
Isolation of factors that affect the SgrS terminator. (A) DNA sequence around the junction of *sgrS-setA*-*lacZ* or *sgrS*Δ*T-setA*-*lacZ*. The inverted repeat sequences of the *sgrS* terminator are indicated by underlining. Nucleotides are numbered from the site corresponding to the 5′ end of *sgrS*. A possible Shine-Dalgarno sequence and the initiation codon of *setA* are boxed. Alanine of the ninth codon in *lacZ* mRNA was connected to arginine of the 10th codon of *setA* mRNA. (B) Lactose MacConkey ampicillin indicator plate with TM1013 (*Cp17*-*sgrS*-*setA*-*lacZ* Δ*sgrS*) transformed by the pHDB3 vector control or a plasmid from each group, for groups 1 to 9. Genes contained in each group and total numbers of plasmids isolated are shown in the table on the right. The pRED-number in parentheses refers to the serial number of isolated clones. (C) β-Galactosidase assay to evaluate the activity of the isolated plasmids on the transcriptional readthrough at the SgrS terminator. TM1013 (*Cp17*-*sgrS*-*setA*-*lacZ* Δ*sgrS*) and TM1014 (*Cp17*-*sgrS*Δ*T*-*setA*-*lacZ* Δ*sgrS*) were transformed by the pHDB3 vector control or a plasmid from each group, for groups 1 to 9. Colonies from a lactose MacConkey (Amp) plate were suspended in fresh chilled LB medium and assayed for β-galactosidase as described in Materials and Methods. Groups that were chosen for further study are indicated with an asterisk. The results are averages of three independent experiments, with error bars representing the standard deviations. The levels of values for *sgrS*Δ*T-setA*-*lacZ* relative to those in *sgrS-setA*-*lacZ* are shown below the graph (e.g., 1.6 for the vector control).

To monitor transcriptional readthrough at sRNA terminators, we focused on the *sgrS-setA* operon. The *setA* gene is cotranscribed with *sgrS* from a transcript activated by the SgrR transcription factor in response to glucose-phosphate stress. The *setA* translational initiation site is 27 bp downstream of the 3′ end of the *sgrS* terminator. We constructed *lacZ* translational fusions to *sgrS-setA* and a variant lacking the *sgrS* terminator at the chromosomal *lacZ* locus as reporter genes to monitor the readthrough (Fig. 1A). The fusions were driven by a constitutive synthetic promoter, Cp17 (25), so that transcription was independent of the regulatory circuit for the glucose-phosphate stress response.

To screen for genetic factors that affect the SgrS terminator, we used a pBR322-based multicopy plasmid library (26, 27). The plasmids contained fragments from the E. coli genome ranging from 1 to 5 kb in size. The vector control (pHDB3) in the *sgrS-setA-lacZ* strain gives a light red colony on lactose MacConkey indicator plates, which could result from low-level transcriptional readthrough at the SgrS terminator (Fig. 1B, vector). By screening ~70,000 individual transformants on lactose MacConkey (Amp) plates, we isolated 47 clones that gave redder colonies. These were candidates for plasmids in which the inserted DNA fragment led to overexpression of a factor that attenuated termination or otherwise increased amounts or activity of the SetA-LacZ fusion protein. Some plasmids gave white colonies, possibly reflecting increased termination, but further analysis has not yet been carried out for those plasmids.

DNA sequences for the ends of each genomic insert were determined and matched with the E. coli genome. Based on the overlapping regions between the inserts, redder clones were classified into nine groups (Fig. 1B, sectors 1 to 9, corresponding to groups 1 to 9). Plasmids selected from each group and the vector plasmid were used to transform the original fusion strain and a control fusion with the *sgrS* terminator deleted (Fig. 1A, *sgrS*Δ*T-setA-lacZ*), and we assayed for β-galactosidase activity. The absence of the SgrS terminator increased β-galactosidase activity ~1.6- to 1.8-fold, indicating that the SgrS terminator functions as a partial block for downstream *setA* expression, presumably by transcription termination (Fig. 1C, open bar for pHDB3 compared to filled bar). All plasmids led to modest, although reproducible, increases in expression of the *setA-lacZ* fusion containing the SgrS terminator (Fig. 1C, open bars), consistent with what was seen on the lactose MacConkey (Amp) plate. We were particularly interested in the factors that affected intrinsic termination. Therefore, those plasmids which led to very similar expression levels of the reporter with (wild type [WT]) and without (ΔT) the terminator were chosen for further study (ΔT/WT ratio, ≤1). These included plasmids belonging to groups 1, 2, 5, and 6 (Fig. 1C, indicated with an asterisk). The ΔT/WT ratio reflected both a decreased level of activity in the ΔT fusion (Fig. 1C, filled bars) and an increased level in the WT fusion for all four plasmids. The lower expression levels for the ΔT fusion suggested that these plasmids affect transcription or mRNA stability as well as any effects on termination. However, none of the plasmids affected the expression of a *Cp17-lacZ* control fusion, in which the *lacZ* gene was directly fused to the Cp17 promoter (see Fig. S1 in the supplemental material), suggesting that any effects on transcript levels are not general.

10.1128/mbio.02371-22.1 FIG S1 β-Galactosidase assay of identified plasmids with a control fusion. Strain BA882 containing a plasmid from each group was grown and assayed as described for Fig. 1C. The results are an average of three independent experiments, with error bars representing the standard deviations. Relative levels of values were calculated, with the value of the pHDB3 vector control set to 1. Download FIG S1, PDF file, 0.2 MB. Copyright © 2022 Morita et al. 2022 Morita et al. https://creativecommons.org/licenses/by/4.0/ This content is distributed under the terms of the Creative Commons Attribution 4.0 International license.

### Multicopy attenuator of SgrS terminator.

Genomic inserts in plasmids usually contain two to four genes. In four groups, alignment of inserts allowed the identification of common complete genes within each group: *cyaR-ogrK-yegR* (group 1), *cspD* (group 2), *yaeP-rof* (group 5), and *ygjH* (group 6) (Fig. S2). To define the genes that were needed for the observed phenotype of increased fusion activity, candidate genes from groups 2, 5, and 6 were recloned in expression plasmids and reintroduced to the reporter strain. In group 1, *cyaR*, encoding a sRNA, was responsible for the activity (Fig. S3A, sectors 1 and 2) and is discussed further below.

10.1128/mbio.02371-22.2 FIG S2 Genetic organization and mapping of the inserts for groups 1, 2, 5, and 6. The sequencing results were mapped with the E. coli K-12 genome using a BLASTn search (https://ecocyc.org/ECOLI/blast.html). For inserts that were mapped to two distant regions (pRED51, -21, -38, -47, and -23), only the relevant region is shown (arrows). Because one side of the sequencing in pRED27 matched with the region from 2,172,082 to 2,170,999, it was included in group 1. Download FIG S2, PDF file, 0.1 MB. Copyright © 2022 Morita et al. 2022 Morita et al. https://creativecommons.org/licenses/by/4.0/ This content is distributed under the terms of the Creative Commons Attribution 4.0 International license.

10.1128/mbio.02371-22.3 FIG S3 Identification of critical regions in isolated plasmids. Lactose MacConkey-ampicillin indicator plates with the indicated strains and plasmids were incubated overnight at 37°C. Download FIG S3, PDF file, 0.3 MB. Copyright © 2022 Morita et al. 2022 Morita et al. https://creativecommons.org/licenses/by/4.0/ This content is distributed under the terms of the Creative Commons Attribution 4.0 International license.

Expression of *cspD* from group 2 was sufficient for the redder phenotype (Fig. S3B). The *cspD* gene encodes a cold shock protein (CSP) and, unlike other CSPs, is expressed in stationary phase (28). A previous study demonstrated that CspD bound to both RNA and single-stranded DNA and inhibited plasmid replication *in vitro*; overproduction of CspD reduced cell viability (29).

The genes *yaeP* and *rof* from group 5 were initially tested together because the stop codon for *yaeP* overlaps the first few codons of *rof*, suggesting *yaeP* may play a role in modulation of *rof* expression. The expression of *yaeP-rof* was sufficient for the redder phenotype (Fig. S3B). To determine which gene is involved in the phenotype, plasmids carrying each gene driven by an isopropyl-β-d-thiogalactopyranoside (IPTG)-inducible promoter were tested. While the expression of *rof* inhibited colony formation, presumably because of high expression in response to lactose, the expression of *yaeP* affected neither colony formation nor its color (Fig. S3B). These results suggest that the *rof* gene is necessary for the original phenotype. The *rof* gene encodes a factor that prevents the termination factor Rho from binding to single-stranded DNA (30).

Expression of *ygjH* from group 6 was sufficient to increase fusion expression (Fig. S3B). The *ygjH* gene encodes a unique protein that is a homolog to a structure-specific tRNA binding protein, Trbp111, in Aquifex aeolicus (31); however, the function of this protein *in vivo* remains to be studied.

### Indirect role of CyaR on SgrS termination.

sRNAs are classified into two classes according to which surface of the Hfq RNA chaperone they bind: class I sRNAs bind to the proximal face and rim surfaces of the Hfq hexamer, while class II sRNAs bind the proximal and distal faces of the Hfq hexamer (32). A unique characteristic of class II sRNAs is that they act as efficient titrators of Hfq (33, 34), thus raising the possibility that CyaR, a class II sRNA, attenuates the SgrS terminator through this titrator effect. However, other major class II sRNAs (ChiX and McaS) did not increase reporter expression (Fig. S3A). The increased reporter expression seen with CyaR overexpression was lost in the absence of *hfq* or in strains expressing an *hfq* mutant in the distal face (with a Y-to-D change at position 25 [Y25D]) or the rim surface (R16A) of the Hfq hexamer (Fig. S3C). While deletion of *hfq* and the distal face mutant should reduce the amount of CyaR produced from the plasmid, the rim surface mutant should interfere with CyaR function but not lead to lower CyaR levels (32). These results strongly suggest that the CyaR activity on the SgrS terminator is indirect, possibly through regulation of one or more of its target mRNAs. Supporting this, point mutants in the pairing region of CyaR were tested (35). A44T and triple mutant G38C G39A A40T eliminated CyaR activity on the reporter; in contrast, T47A did not (Fig. S3D). We considered the possibility that *cspD*, *rof*, or *ygjH* was the CyaR target; if so, we would have expected CyaR to positively regulate the gene, to mimic the effect of overproduction. However, CyaR was still able to upregulate the reporter in strains carrying deletions of each factor (Fig. S3E). No further work was done to identify the CyaR target(s) that are responsible for this phenotype; more than 200 possible CyaR targets have been identified with RNA sequencing (RNA-seq)-based approaches (36–39). No evidence was found for a direct interaction of CyaR with SgrS or *setA*.

### Double terminator system confirms attenuator roles of CspD, Rof, and YgjH.

**FIG 2:**
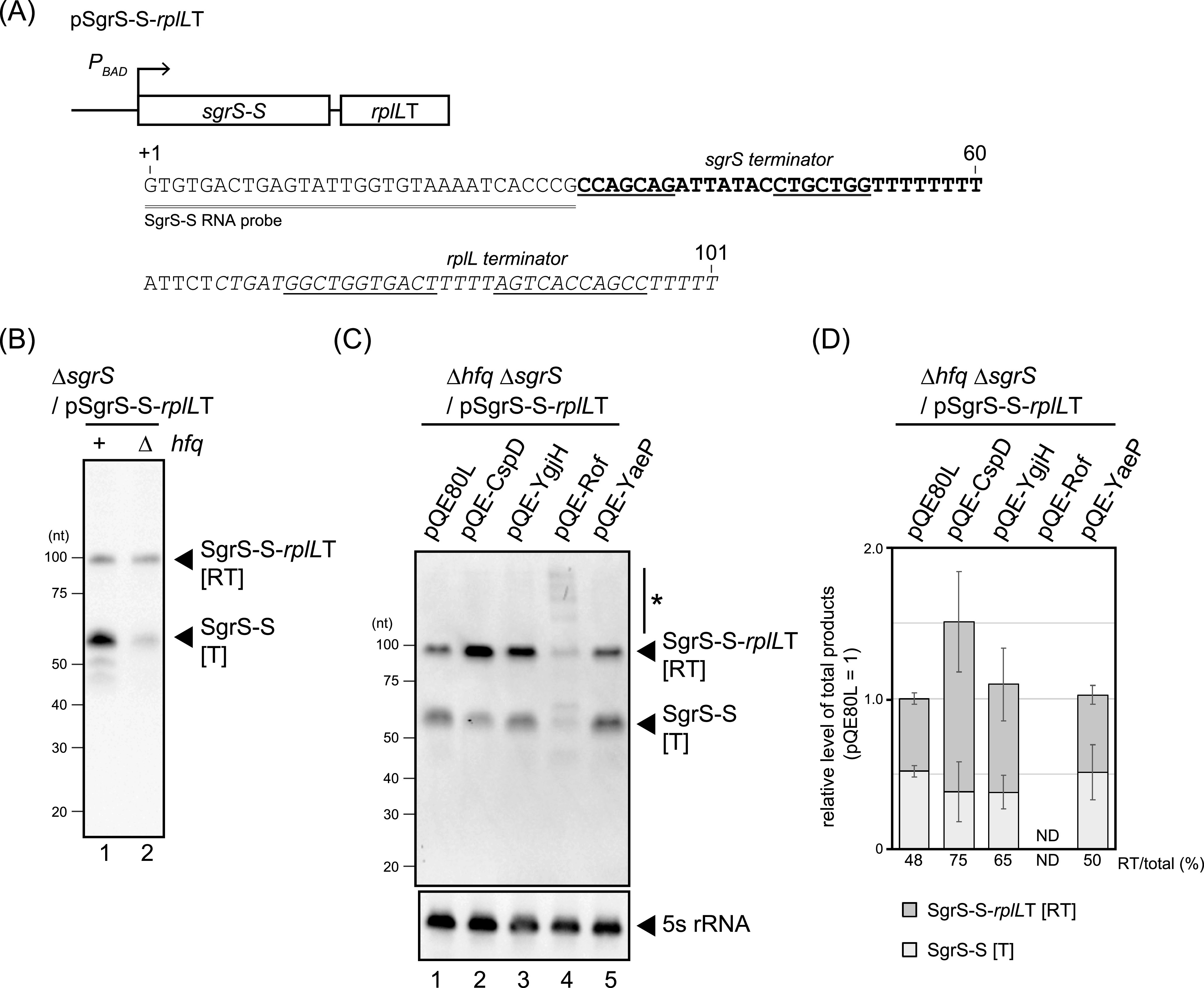
Evaluation of readthrough at the SgrS terminator with a double terminator system. (A) DNA sequence of the *sgrS-S-rplL*T hybrid gene. The sequence of the *sgrS* terminator is shown in bold. The sequence of the downstream terminator derived from *rplL* is shown in italics. The inverted repeat sequences within the terminators of *sgrS* and *rplL* are indicated by underlining. pAraX, used as the expression vector of the hybrid gene, is a low-copy-number plasmid derived from pMW218. The SgrS-S RNA probe used for the Northern blotting detected the double-underlined sequence. (B) Analysis of transcriptional readthrough with the double terminator system. Total RNA was isolated from TM542 (*hfq*^+^ Δ*sgrR-sgrS*) and TM772 (Δ*hfq* Δ*sgrR-sgrS*) cells harboring pSgrS-S-*rplL*T grown in LB-kanamycin medium in the presence of 0.4% arabinose to an OD_600_ of 0.5. The RNA sample (5 μg) was subjected to Northern blotting using the SgrS-S probe. (C) Factors affecting the termination of *sgrS*. TM772 (Δ*hfq* Δ*sgrR-sgrS*) cells harboring the indicated plasmids were grown in LB-kanamycin, ampicillin medium. At an OD_600_ of 0.2, 0.2 mM IPTG was added to cultures to induce attenuation factors, and incubation was continued for 60 min. Then, 0.4% arabinose was added to cultures to induce *sgrS-S*-*rplL*T, and incubation was continued for 10 min. Total RNA was prepared, and the RNA samples (5 μg and 1 μg) were subjected to Northern blotting using probes of SgrS-S and 5S rRNA, respectively. The asterisk indicates transcripts extending beyond the second terminator. (D) Quantitation of the Northern blotting data. Upper dark gray bars indicate the readthrough transcript [RT], lower light gray bars indicate the terminated product [T], and the total bar indicates the sum of T and RT. Relative levels of RNA were calculated, with the RNA sample of the vector control set to 1. Percentage of the readthrough transcript [RT] relative to total is shown at the bottom. The results are averages of three independent experiments, with error bars representing the standard deviations.

The β-galactosidase assay used above suggested that the identified genes affect SgrS termination efficiency (Fig. 1C). To directly visualize their activities on SgrS termination, we investigated transcriptional readthrough at the SgrS terminator using our previously described double terminator system (22). This system uses a low-copy-number plasmid expressing a hybrid gene in which the stable transcription terminator of *rplL* is placed downstream of the minimal functional region of SgrS (denoted as SgrS-S) (Fig. 2A). Northern blot analysis with a probe within SgrS-S detected both the transcript ending at the SgrS terminator ([T]: SgrS-S) and a longer readthrough transcript, extending to the second terminator ([RT]: SgrS-S-*rplL*T) (Fig. 2B).

Hfq stabilizes the transcript that terminates at the end of *sgrS*, but not the readthrough transcript that ends at the *rplL* terminator, whose poly(U) tail of five is too short to bind Hfq (22). To avoid differences in RNA accumulation due to Hfq-dependent stabilization of transcripts that terminate at the end of *sgrS*, an *hfq* mutant was used to evaluate the readthrough. We first confirmed the previous observation (22) that, in the Δ*hfq* background, comparable levels of [RT] and [T] were detected (Fig. 2B).

The identified genes were individually cloned into the pQE80L vector, under the control of an IPTG-inducible promoter. In this study, we induced expression of each factor by the addition of IPTG, then induced the *sgrS-S-rplL*T hybrid gene by the addition of arabinose. Northern blot analysis of the pQE80L control sample and its quantitation show that [RT] accounted for 48% of the total signal (Fig. 2C and D). For cells overexpressing CspD and YgjH, the ratio of [RT] to total increased to 75% and 65%, respectively, consistent with these factors enhancing readthrough at the *sgrS* terminator. Additionally, the total amount of both transcripts in the CspD overexpression strain increased 1.5-fold compared to the vector control, with much of this increase attributable to more of the [RT] transcript (Fig. 2D). Rof produced a range of aberrant transcripts, including many that were longer than [RT] (Fig. 2C, indicated with an asterisk), making it difficult to quantitate the levels. These results corroborated that *rof* is the gene responsible for the phenotype of the original group 5 plasmid. YaeP did not affect readthrough, consistent with its lack of phenotype in the plate assay. CspD, YgjH, and Rof are hereafter referred to as attenuation factors; when overproduced, they reduced intrinsic termination, directly or indirectly.

### Attenuation factors affect terminators of other sRNAs.

**FIG 3:**
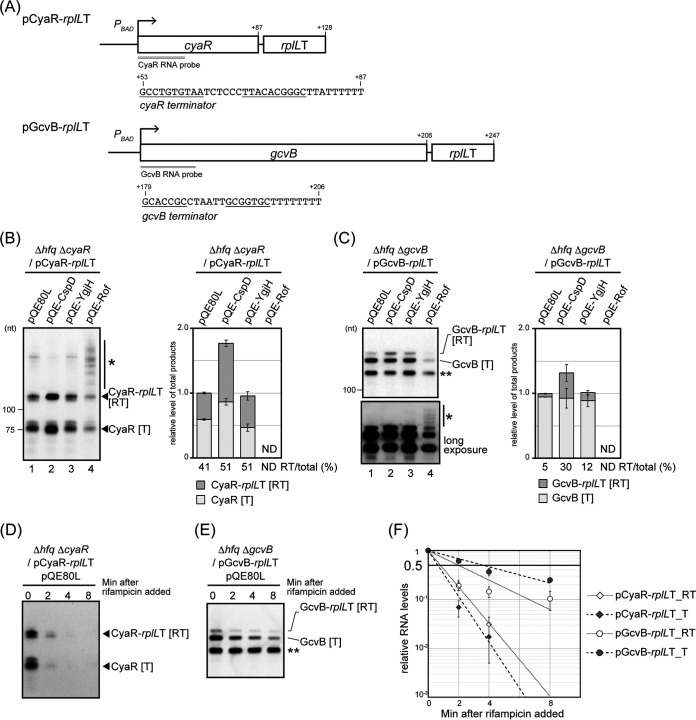
Effect of attenuation factors on the intrinsic termination of sRNAs CyaR or GcvB. (A) Diagrams of the *cyaR-rplL*T hybrid gene and the *gcvB-rplL*T hybrid gene. The DNA sequences of the terminators of these sRNAs are shown. The CyaR and GcvB RNA probes used for the Northern blotting detected the double-underlined regions. (B and C) Analyses of transcriptional readthrough at the terminators of CyaR and GcvB. TM1180 (Δ*hfq* Δ*cyaR*) cells (B) or TM1191 (Δ*hfq* Δ*gcvB*) cells (C) harboring the indicated plasmids were used. At an OD_600_of 0.2, 0.2 mM IPTG was added to cultures to induce attenuation factors, and incubation was continued for 60 min. Then, 0.4% arabinose was added to cultures to induce *sRNA*-*rplL*T and incubation was continued for 10 min. Total RNA was prepared and the RNA sample (2 μg) was subjected to Northern blotting using probes for CyaR (B) or GcvB (C). The asterisk indicates transcripts extending beyond the second terminator. In panel C, the shorter band (indicated with **) is the transcript derived from the mutated chromosomal copy of *gcvB*, in which the internal base-pairing region was deleted on the chromosome (73). The graphs show quantitation of the Northern blot data. The results are averages of three independent experiments, with error bars representing the standard deviations. (D to F) Stabilities of sRNAs and their readthrough products. TM1180 (Δ*hfq* Δ*cyaR*) cells (D) or TM1191 (Δ*hfq* Δ*gcvB*) cells (E) harboring the indicated plasmids were grown under the same condition as in panels B and C. Then, rifampin (250 μg mL^−1^) was added, and total RNA was prepared at the indicated times after the addition of rifampin. The RNA sample (2 μg) was subjected to Northern blotting using the probe of CyaR (D) or GcvB (E). (F) Each half-life was estimated by a scatterplot. The results represent the means of three independent measurements, and error bars indicate the standard deviations. For CyaR, data from 0-, 2-, and 4-min samples were used for the calculation. Open diamonds, [RT] of CyaR-*rplL*T; closed diamonds, [T] of CyaR; open circles, [RT] of GcvB-*rplL*T; closed circles, [T] of GcvB.

To further explore the activities of the attenuation factors, we investigated their effects on two other Hfq-binding sRNAs, CyaR and GcvB, which are easily detected during exponential growth phase in LB medium (40). Genes encoding these two sRNAs contained the sequence of a predicted typical intrinsic terminator at the end (Fig. 3A). We constructed double terminator genes (*cyaR*-*rplL*T and *gcvB*-*rplL*T) on the plasmid, as we had with SgrS-S, to detect readthrough transcripts (Fig. 3A). Each plasmid was introduced into the strain deleted for both *hfq* and the chromosomal copy of the sRNA. Northern blot analyses demonstrated that both hybrid genes produced longer RNA > RNAs corresponding to the product extended to the second terminator [RT] along with the expected sRNAs [T] (Fig. 3B and C, lane 1), indicating that readthrough occurred at the terminators of these two sRNAs.

The [RT] of *cyaR*-*rplL*T was 41% of the sum of [T] and [RT], similar to that seen for SgrS-S. Note that while CyaR was one of the factors found to stimulate readthrough for SgrS, this was not observed in the absence of *hfq* (Fig. S3C). Therefore, we interpreted the readthrough seen here as that intrinsic to the CyaR terminator. For CyaR, the half-lives of the [T] and [RT] were 0.6 min and 0.8 min, respectively (Fig. 3D and F), suggesting that the calculated [RT] (41%) at the CyaR terminator reflects the efficiency of readthrough rather than any major difference in mRNA stability.

The apparent readthrough for GcvB was 5%, significantly less than that for SgrS or CyaR (Fig. 3C). However, in this case, the [T] transcript had a half-life of 3.6 min, while the [RT] transcript had a half-life of 2.0 min (Fig. 3E and F). This difference in stability would lead to underestimating the readthrough efficiency, but probably not sufficiently to account for the apparent very different efficiency of readthrough for the GcvB terminator. Overall, these results suggest that readthrough of sRNA terminators may be a general phenomenon. The basis for the differences in termination efficiencies remains to be studied.

The three protein attenuators were introduced into strains carrying the CyaR and GcvB hybrid plasmids, and Northern blot analyses were done to evaluate their ability to affect termination efficiency (Fig. 3B and C). For CyaR, the overproduction of either CspD or YgjH increased the ratio of [RT] to a total of 51%. For GcvB, the ratio of [RT] to total increased to 30% or 12% in the CspD or YgjH overproduction strain, respectively. Additionally, the total transcripts of CyaR or GcvB in the CspD overproduction strain increased 1.8-fold or 1.3-fold, respectively, in comparison to the vector control. In the Rof overproduction strain, longer aberrant transcripts of CyaR-*rplL*T were produced (Fig. 3B, indicated with an asterisk). For GcvB, transcripts of [T] and [RT] were drastically reduced in the Rof overproduction strain, and the longer aberrant transcripts were visible after a long exposure (Fig. 3C, bottom panel, indicated with an asterisk). Together, these results indicated that the attenuation factors isolated for their effect on SgrS also acted as attenuators on the terminators of CyaR and GcvB.

### Attenuation factors reduce regulation by SgrS.

**FIG 4:**
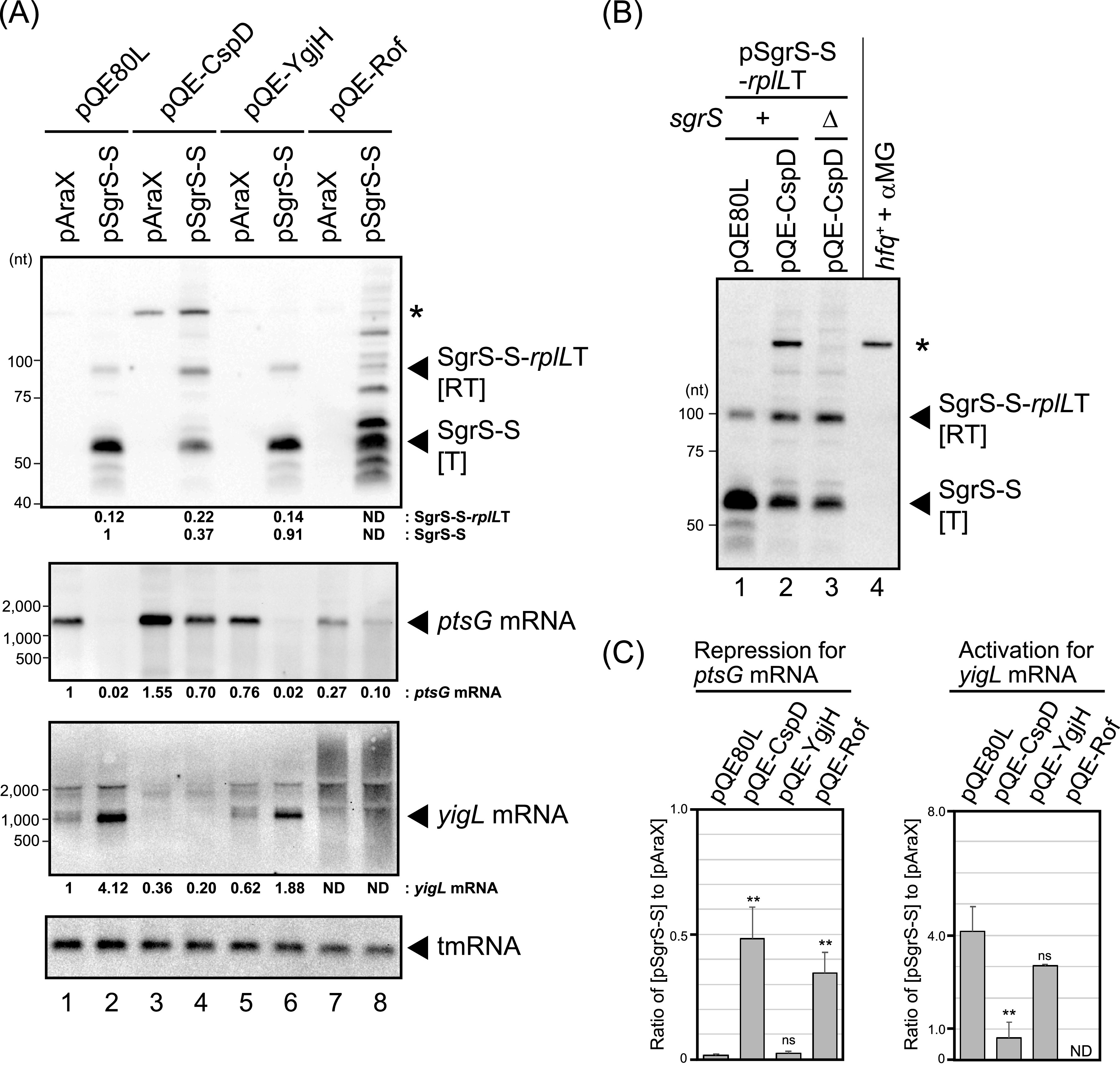
Attenuation factors abrogate the regulation by SgrS. (A) IT1568 cells harboring the indicated plasmids were used. Overnight cultures were diluted 1,000-fold into LB-kanamycin, ampicillin medium in the presence of 0.1 mM IPTG and 0.4% arabinose. At an OD_600_ of 0.5, total RNA was prepared. RNA samples (5 μg, 4 μg, 4 μg, and 1 μg) were subjected to Northern blotting using probes of SgrS-S, *ptsG* mRNA, *yigL* mRNA, and tmRNA, respectively. Relative levels of each RNA compared to the vector control were calculated with an average of three independent experiments. Representative Northern blots are shown. (B) Effect of CspD overproduction on the expression of endogenous SgrS. For samples of lanes 1 to 3, IT1568 (lanes 1 and 2) or TM542 (lane 3) cells harboring the indicated plasmids were used. Overnight cultures were diluted 1,000-fold into LB-kanamycin, ampicillin medium in the presence of 0.1 mM IPTG and 0.4% arabinose. At an OD_600_ of 0.5, total RNA was prepared. For the sample of lane 4, IT1568 cells were grown in LB medium. At an OD_600_ of 0.5, 0.1% α-MG was added to cultures and incubation was continued for 10 min before isolating total RNA. Five micrograms (lanes 1 to 3) and 0.5 μg (lanes 4) of RNA samples were subjected to Northern blotting using the SgrS-S probe. Endogenous SgrS is indicated by an asterisk. (C) Effects of attenuation factors on the regulation by SgrS-S of *ptsG* and *yigL* mRNAs. Ratios of target mRNA levels in the pSgrS-S strains to those in the pAraX strains were calculated. Statistical significance was calculated using an unpaired two-tailed Student's *t* test. ns, not significant; **, *P* < 0.01.

The ability of the attenuation factors to perturb sRNA termination suggested they might also interfere with sRNA signaling, by reducing the amount of the properly terminated sRNA. To test this, we analyzed two target mRNAs of SgrS, *ptsG* and *yigL*, encoding the membrane component of the major glucose transporter and a HAD-like phosphatase, respectively. Note that these experiments, unlike those above, were done in an *hfq*^+^ host, where SgrS-S is more stable than the readthrough transcript. This indeed resulted in a relative ratio of the [RT] product to SgrS-S of 11% (Fig. 4A, lane 2), rather that the 48% seen in the absence of Hfq (Fig. 2). The ability of SgrS-S to regulate *ptsG* and *yigL* in the *hfq*^+^ host was examined as well (Fig. 4A, lower panels). The regulation by SgrS of *ptsG* or *yigL* mRNAs is highly efficient (11); SgrS represses *ptsG* mRNA, whereas it activates *yigL* mRNA. In the pQE80L control sample, SgrS-S expression caused a reduction of *ptsG* mRNA and an increase in *yigL* mRNA (Fig. 4A, lanes 1 and 2), indicating that SgrS-S functions under this condition.

Each attenuation factor was overexpressed, and the levels of SgrS-S and the [RT] product relative to the vector control were determined (Fig. 4A, lanes 4, 6, and 8). CspD overproduction led to an increase in [RT] and a decrease in levels of SgrS-S (Fig. 4A, lane 4). A longer transcript was also detected (Fig. 4A, lanes 3 and 4, indicated with an asterisk). This transcript was likely expressed from the chromosomal copy of *sgrS*, as it was absent in an Δ*sgrS* background (Fig. 4B), although its levels were significantly less than those fully induced by the cognate stress (Fig. 4B, lane 4; the RNA sample was loaded at 1/10 the level of the other lanes). Further analysis has not yet been conducted for this SgrS induction by CspD. Consistent with a decrease in SgrS-S levels, CspD overproduction greatly reduced both the repression of *ptsG* mRNA and the activation of *yigL* mRNA (Fig. 4A, lanes 3 and 4, and 4C; bars for pQE-CspD compared to those for pQE80L).

YgjH overproduction led to slight effects on levels of both the [RT] product and SgrS-S. There was also no significant effect on the regulation by SgrS-S (Fig. 4A, lanes 5 and 6, and 4C; bars for pQE-YgjH compared to those for pQE80L). These results indicated a lower effect than what would be predicted from the plate assays (Fig. 1). It is possible that YgjH action may be favored by cell growth on the plate or what is contained in MacConkey lactose agar.

Rof overproduction exhibited perturbation of the *sgrS-S-rplL*T transcription, as observed in the Δ*hfq* background (Fig. 2C). Rof overproduction partially reduced the regulation of *ptsG* (Fig. 4A, lanes 7 and 8, and 4C, bars for pQE-Rof compared to those for pQE80L). The *yigL* transcripts seemed to be directly affected by Rof, in the absence of SgrS-S expression (Fig. 4A, lane 7). Rof also caused a reduction in the *ptsG* mRNA in the absence of SgrS-S, implying that Rof efficiently inhibited Rho activity, resulting in general perturbation of transcription.

### Rof and biocyclomycin implicate Rho in SgrS-S transcription.

The results above showed a clear effect of the attenuation factors on intrinsic termination for sRNAs, including the surprising finding that Rof, known as an inhibitor of Rho, had significant effects on the tested sRNA terminators. Rho typically binds to RNA at a *rut* site that is characterized by a single-stranded C-rich/G-poor RNA with a ribosome-free length of 60 to 90 nucleotides (nt), causing transcription termination further downstream of this entry site (reviewed in reference 41). SgrS-S is 60 nt in length and ribosome-free, as it lacks the small open reading frame (ORF) in the 5′ portion of SgrS. The only C-rich stretch is short, and just before the terminator, likely not sufficient for rho termination (see the sequence of SgrS-S in Fig. 2A).

**FIG 5:**
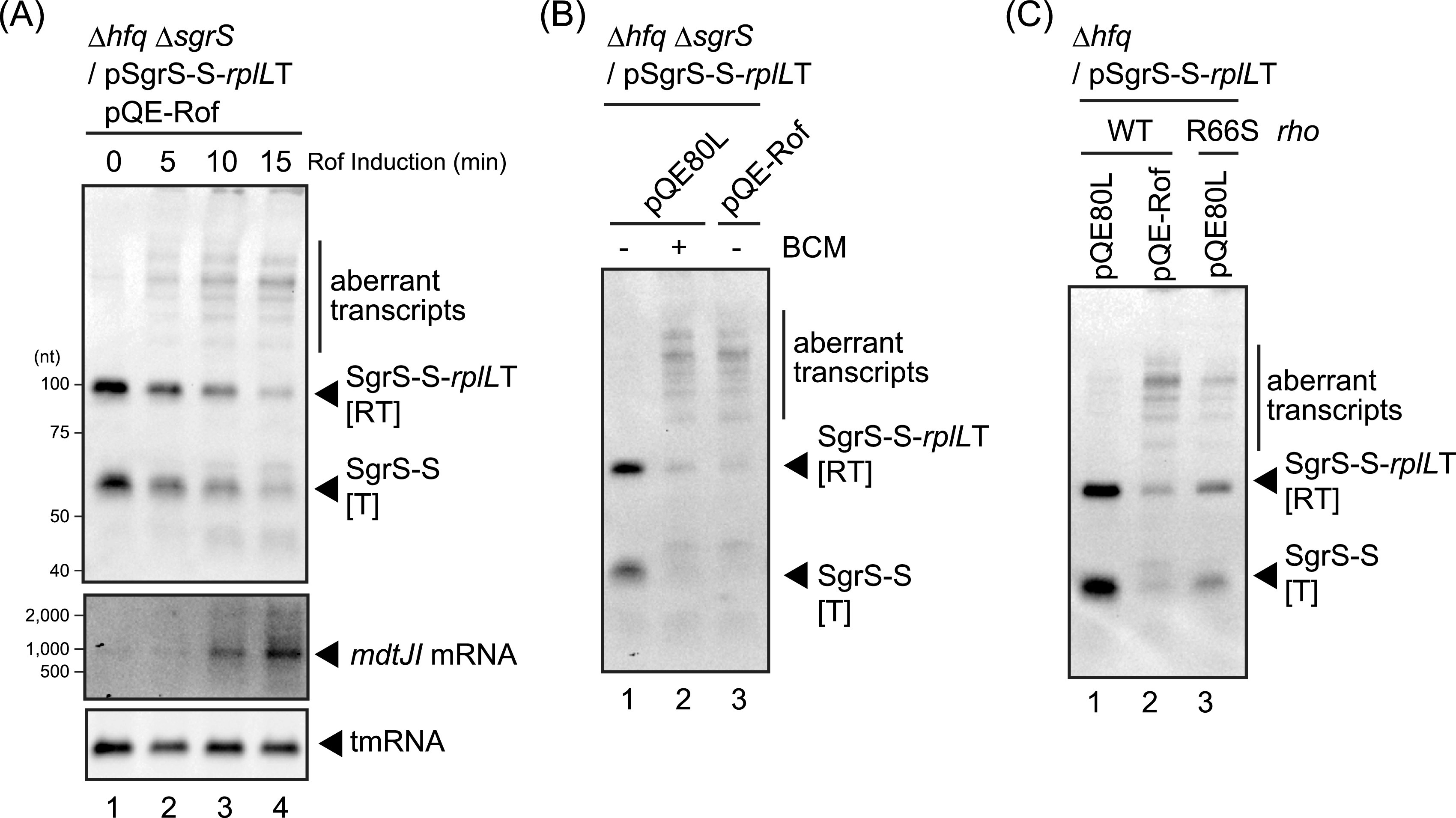
Roles of Rof in the transcription of *sgrS-S-rplL*T. (A) Rapid effect of Rof on *sgrS-S-rplL*T transcription. TM772 (Δ*hfq* Δ*sgrR-sgrS*) cells harboring pSgrS-S-*rplL*T and pQE-Rof were grown in LB-kanamycin, ampicillin medium. At an OD_600_ of 0.5, 0.4% arabinose was added to cultures to induce *sgrS-S*-*rplL*T and incubation was continued for 10 min. Then, 0.2 mM IPTG was added to cultures to induce *rof*, and total RNA was prepared at the indicated times after the addition of IPTG. The RNA samples (5 μg, 2 μg, and 1 μg) were subjected to Northern blotting using probes for SgrS-S, *mdtJ* mRNA, and tmRNA, respectively. (B) Effect of BCM on *sgrS-S-rplL*T transcription. TM772 (Δ*hfq* Δ*sgrR-sgrS*) cells harboring pSgrS-S-*rplL*T and the indicated plasmids were used. At an OD_600_ of 0.2, 0.2 mM IPTG was added to cultures and incubation was continued for 60 min. For BCM treatment, 50 μg mL^−1^ BCM was added to cultures 30 min after the addition of IPTG, and incubation was continued for 30 min. Arabinose at 0.4% was added to cultures and incubation was continued for 10 min. Total RNA was prepared and the RNA sample (5 μg) was subjected to Northern blotting using the SgrS-S probe. (C) Effect of a point mutant in *rho* on *sgrS-S-rplL*T transcription. TM587 (Δ*hfq*) and TM1205 (Δ*hfq rhoR66S*) harboring pSgrS-S-*rplL*T and the indicated plasmids were used. At an OD_600_ of 0.2, 0.2 mM IPTG was added to cultures and incubation was continued for 60 min. Then, 0.4% arabinose was added to cultures and incubation was continued for 10 min. Total RNA was prepared, and the RNA sample (5 μg) was subjected to Northern blotting using the SgrS-S probe.

Rof is known to act on Rho, implying a role for Rho in transcription and termination of the sRNA genes. We used additional approaches with the *sgrS-S-rplL*T gene in the Δ*hfq* background to test if Rho is needed for proper sRNA termination. First, we compared the kinetics of Rof induction on sRNAs to the effect on a known Rho-dependent termination event. *mdtJI* mRNA is known as a target of Rho-mediated premature termination (42). Therefore, an increase in *mdtJI* mRNA would be expected by Rof inhibition of Rho. Indeed, a time course experiment consistently showed that Rof induction resulted in the rapid production of *mdtJI* mRNA (Fig. 5A), indicating that induction of Rof from the plasmid was sufficient to inhibit Rho activity. Strikingly, the aberrant transcripts derived from the *sgrS-S-rplL*T gene appeared as rapidly as did the *mdtJI* mRNA (Fig. 5A). Consistent with this, bicyclomycin (BCM), which is a Rho inhibitor, also led to the production of the aberrant transcripts (Fig. 5B, compare lane 2 with lane 1). We then examined the effect of a point mutation in *rho* (R66S) on *sgrS-S-rplL*T transcription. The *rho* R66S mutation disrupts the RNA binding site and causes a considerable defect in Rho activity (42, 43). Northern blot analysis showed that aberrant SgrS-S transcripts and a reduction in SgrS-S were also observed in the *rho* R66S background (Fig. 5C, compare lane 3 with lane 1). These results strongly suggest that Rho participates in *sgrS-S* transcription and its termination in a direct manner and raise the question of how general Rho participation in sRNA termination is.

### Lack of attenuation factors did not alter readthrough under the conditions tested.

The analysis described above demonstrated that the attenuation factors, when overproduced, interfere with efficient termination of sRNAs, each with a somewhat different phenotype. We tested the phenotype of cells deleted for a single attenuation factor in two ways. First, we asked if deleting one factor interfered with the ability of the overproduced factors to perturb SgrS-S termination. Northern blot analyses showed that the increase in readthrough transcripts by factor overproduction was not significantly affected by the individual mutations (Fig. S4A), demonstrating that the activity of each factor was independent of the other factors.

10.1128/mbio.02371-22.4 FIG S4 Effect of mutations in each attenuator on the transcriptional readthrough at the SgrS terminator. (A) TM587 (Δ*hfq*), TM1111 (Δ*hfq* Δ*cspD*), TM1112 (Δ*hfq* Δ*rof*), and TM1113 (Δ*hfq* Δ*ygjH*) cells harboring the indicated plasmids were grown in LB-kanamycin, ampicillin medium. When the OD_600_ reached 0.2, 0.2 mM IPTG was added to cultures to induce attenuators and incubation was continued for 60 min. Then, 0.4% arabinose was added to cultures to induce *sgrS-S*-*rplL*T and incubation was continued for 10 min. Total RNA was prepared and the 5 μg of RNA sample was subjected to Northern blotting using the SgrS-S probe. (B) TM587 (Δ*hfq*) cells harboring the indicated plasmids were grown in LB-kanamycin, ampicillin medium. At an OD_600_ of 0.5, 0.4% arabinose was added to cultures and incubation was continued for 10 min. Then, 250 μM 2,2′-dipyridyl (Dip) was added to cultures and incubation was continued for 10 min. The RNA sample (5 μg) was subjected to Northern blotting using the SgrS-S probe. (C) TM587 (Δ*hfq*) and TM772 (Δ*hfq* Δ*sgrS*) cells were grown in LB medium. At an OD_600_ of 0.5, 0.1% α-MG or 250 μM Dip was added to cultures and incubation was continued for 10 min. The RNA sample (5 μg) was subjected to Northern blotting using the SgrS-S probe. Download FIG S4, PDF file, 0.5 MB. Copyright © 2022 Morita et al. 2022 Morita et al. https://creativecommons.org/licenses/by/4.0/ This content is distributed under the terms of the Creative Commons Attribution 4.0 International license.

It was previously observed that the termination of SgrS is enhanced under several stress conditions (22), although the basis for the regulation at the termination step is not yet understood. We thus tested the role of the attenuation factors for this stress effect by exposing the mutant cells to Fe^2+^ starvation by treatment with the iron-chelating compound 2,2′-dipyridyl (Dip), one of the stresses in which the SgrS termination is enhanced. None of the mutations altered the decrease in [RT] for SgrS-S after stress treatment (Fig. S4B), indicating that these factors are not required for the regulation of SgrS termination, at least under the tested condition. An additional transcript appeared only under the Fe^2+^ starvation condition (Fig. S4B). This transcript was not observed in the previous study with a Δ*sgrS* background, suggesting that it is endogenous SgrS. In fact, Fe^2+^ starvation led to modest SgrS expression in an *hfq* mutant, although the induction level was less than that following addition of the known inducer of glucose-phosphate stress, α-methylglucoside (αMG) (Fig. S4C). Whether the accumulation of SgrS after Fe^2+^ starvation was due to increased transcription (for instance, due to activation of the transcription factor SgrR), increased stability of SgrS, or increased termination efficiency of SgrS was not determined, although given the lower termination efficiency at the identical SgrS-S terminator, we favor an increase in transcription. We began this project to identify the factor(s) affecting the changed termination efficiency under stress conditions. However, our results here suggest that those factors are distinct from those found here.

### CspD stabilizes both terminated and readthrough products in an *hfq* mutant.

We chose to focus our further investigation on CspD. It was significantly more effective than YgjH in promoting readthrough under these conditions and did not disrupt transcription to the extent seen with Rof overproduction.

**FIG 6:**
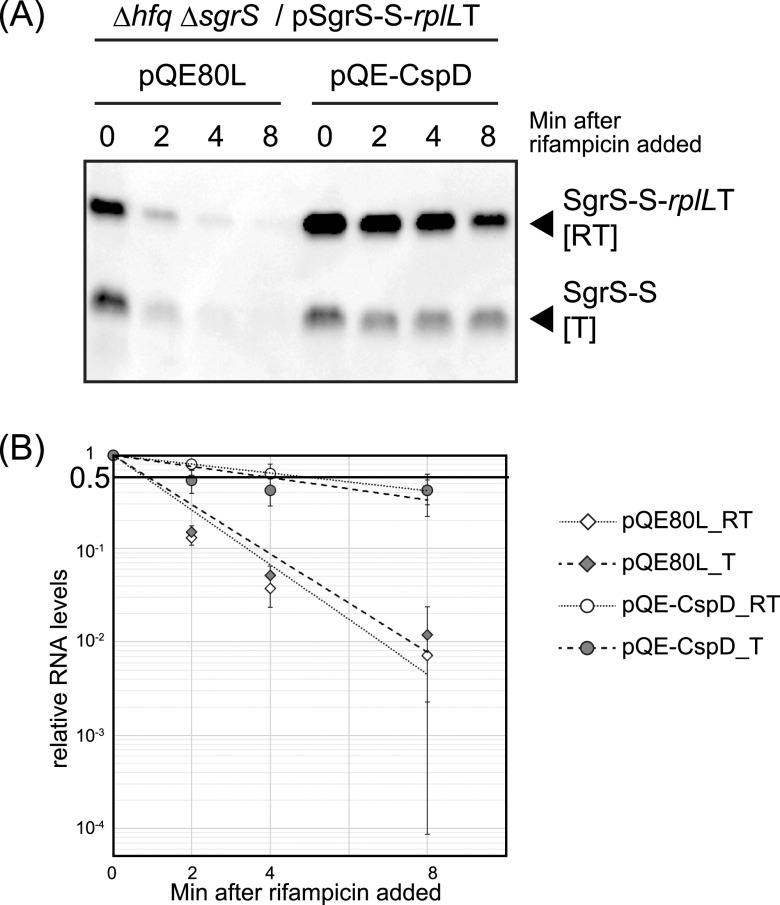
Stabilities of SgrS-S and the readthrough transcript in the CspD overexpression strain. (A) TM772 (Δ*hfq* Δ*sgrR-sgrS*) cells harboring pSgrS-S-*rplL*T and the indicated plasmids were grown in LB-kanamycin, ampicillin medium. At an OD_600_ of 0.2, 0.2 mM IPTG was added to cultures to induce CspD and incubation was continued for 60 min. Then, 0.4% arabinose was added to cultures to induce *sgrS-S*-*rplL*T and incubation was continued for 10 min. Rifampin (250 μg mL^−1^) was added and total RNA was prepared at the indicated times after the addition of rifampin. The RNA sample (5 μg) was subjected to Northern blotting using the SgrS-S probe. A representative Northern blot is shown. (B) Half-lives were estimated by a scatterplot of an average of four independent experiments. Open diamonds, RT in the pQE80L strain; closed diamonds, T in the pQE80L strain; open circles, RT in the pQE-CspD strain; closed circles, T in the pQE-CspD strain.

The total number of SgrS-S transcripts increased upon CspD overexpression in the absence of Hfq (Fig. 2D), suggesting that, in addition to promoting readthrough of the terminator, CspD might stabilize these RNAs. To further examine this, we conducted a rifampin chase experiment in the Δ*hfq* background. In the vector control, turnover of both transcripts was comparably rapid (Fig. 6A), as previously seen (22). Half-lives of SgrS-S [T] and the readthrough transcript [RT] in this experiment were estimated to be 1.1 min and 1.0 min, respectively (Fig. 6B). In the CspD overexpression strain, half-lives of both transcripts were significantly extended, to 5.0 min [T] and 6.3 min [RT], respectively (Fig. 6). Because both SgrS-S [T] and [RT] transcripts were stabilized, we concluded that the *in vivo* efficiency of termination and readthrough at the *sgrS* terminator evaluated in Fig. 2D primarily reflected a change in readthrough efficiency, rather than a differential effect on stability of the two transcripts. It is possible that the modest differences in stability, particularly in the CspD overexpression strain, may affect the precise quantitation of the readthrough efficiency.

The majority of sRNAs are unstable in the absence of Hfq (33). The instability of sRNA RyhB in the absence of Hfq is caused by RNase E-dependent degradation (44). To test whether RNase E is involved in decay of the SgrS-S transcripts, their stabilities were investigated in the *hfq* mutant harboring a temperature-sensitive mutation (*ams-1*) in the *rne* gene. The result indicated that RNase E is likely critical for decay of [RT], whereas degradation of [T] was unaffected by the mutant (Fig. S5A). We additionally showed that the stability of endogenous SgrS was also unaffected by the mutant (Fig. S5B). These results suggest that SgrS, unlike RyhB, is degraded by another decay pathway in the absence of Hfq. Furthermore, the ability of CspD to stabilize the RNase E-dependent degradation of SgrS-S [RT] as well as the RNase E-independent degradation of SgrS-S [T] suggests that it directly acts by binding SgrS-S transcripts rather than by blocking a single RNase.

10.1128/mbio.02371-22.5 FIG S5 Effect of an *rne ts* mutation (*ams1*) on the decay of SgrS-related transcripts in an *hfq* mutant. (A) Effect of *ams1* on the stabilities of SgrS-S and its readthrough product. TM769 (*ams1* Δ*hfq*) cells harboring the indicated plasmids were grown in LB-kanamycin medium at 30°C. At an OD_600_ of 0.5, the temperature was shifted to 42°C and incubation was continued for 10 min. Then, 0.4% arabinose was added to cultures to induce *sgrS-S*-*rplL*T and incubation was continued for 10 min. Rifampin (250 μg mL^−1^) was added, and total RNA was prepared at the indicated time after the addition of rifampin. The RNA sample (5 μg) was subjected to Northern blotting using the SgrS-S probe. Measurements of RNA half-life are shown in the graph to the right. The results represent the means of three independent measurements, and error bars indicate the standard deviations. Open diamonds, [RT] of SgrS-S-*rplL*T; closed diamonds, [T] of SgrS-S. (B) Effect of *ams1* on the stability of endogenous SgrS. TM587 (Δ*hfq*) and TM769 (Δ*hfq ams1*) cells were grown in LB medium at 30°C. At an OD_600_ of 0.5, the temperature was shifted to 42°C and incubation was continued for 10 min. Then, 0.1% α-MG was added to cultures and incubation was continued for 10 min. Rifampin (250 μg mL^−1^) was added and total RNA was prepared at the indicated time after the addition of rifampin. The RNA sample (5 μg) was subjected to Northern blotting using the SgrS-S probe. Download FIG S5, PDF file, 0.2 MB. Copyright © 2022 Morita et al. 2022 Morita et al. https://creativecommons.org/licenses/by/4.0/ This content is distributed under the terms of the Creative Commons Attribution 4.0 International license.

### CspD binds nascent transcripts of *sgrS-S-rplL*T.

**FIG 7:**
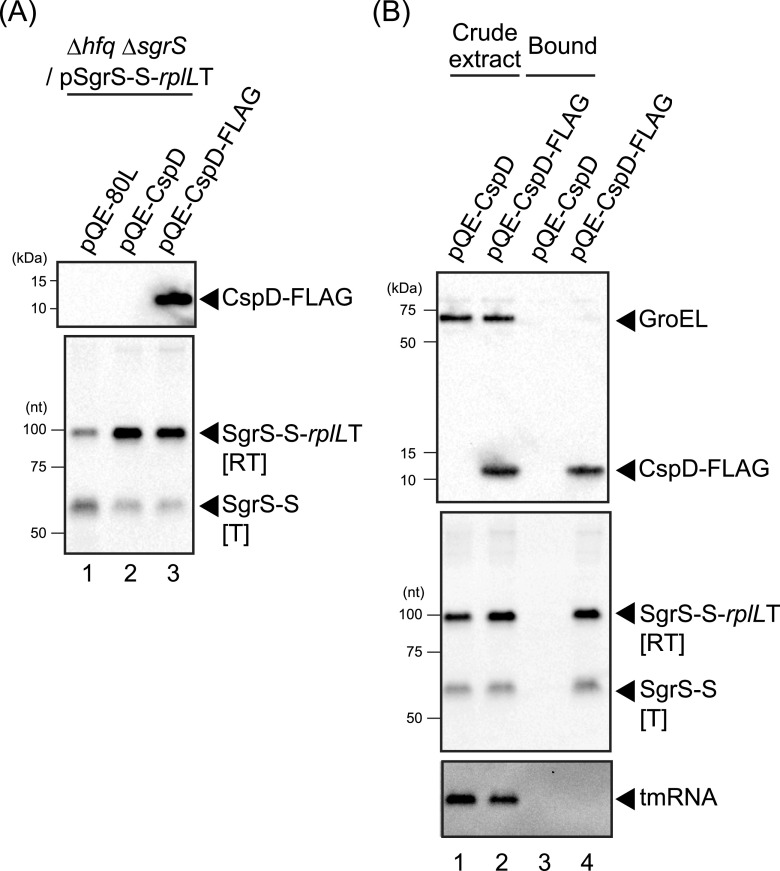
CspD-FLAG binds SgrS-S and the readthrough transcript. (A) Function of FLAG-tagged CspD. TM772 (Δ*hfq* Δ*sgrR-sgrS*) cells harboring pSgrS-S-*rplL*T and the indicated plasmids were grown in LB-kanamycin, ampicillin medium. At an OD_600_ of 0.2, 0.2 mM IPTG was added to cultures to induce *cspD* or *cspD-FLAG*, and incubation was continued for 60 min. Then, 0.4% arabinose was added to cultures to induce *sgrS-S*-*rplL*T and incubation was continued for 10 min. Total protein and RNA were prepared. The protein sample (0.04 units) was subjected to Western blotting using anti-FLAG monoclonal antibody and anti-GroEL polyclonal antibodies. RNA sample (5 μg) was subjected to Northern blotting using the SgrS-S probe. (B) *In vivo* binding of CspD-FLAG to SgrS-S and the readthrough transcript. Crude extract was prepared from TM772 (Δ*hfq* Δ*sgrR-sgrS*) cells harboring pSgrS-S-*rplL*T and the indicated plasmids and then subjected to the pulldown assay using anti-FLAG M2 agarose as described in Materials and Methods.

CspD overproduction increased the total amount of transcripts of all three sRNAs tested (Fig. 2D and Fig. 3B and C) and stabilized both the SgrS-S transcript and the readthrough transcript (Fig. 6). This suggested that CspD likely binds to both sRNA and its readthrough product. To test this possibility, we examined the binding of CspD-FLAG to the SgrS-S transcripts. A FLAG-tag sequence was inserted just upstream of the stop codon of *cspD*; the tagged CspD protein was still functional for attenuation of SgrS-S termination (Fig. 7A). A pulldown assay with anti-FLAG M2 agarose beads demonstrated copurification of both SgrS-S and the readthrough product, but not transfer-messenger RNA (tmRNA), with CspD-FLAG (Fig. 7B). These results indicated that CspD specifically binds to nascent transcripts of the *sgrS-S-rplL*T gene, both attenuating SgrS termination and stabilizing the transcripts.

### Effect of overproducing other CSPs on SgrS termination.

In the E. coli genome, nine genes, *cspA* to *cspI*, are classified as in the CSP family, comprising the CSD (cold shock domain) protein family. The hallmark of these Csps is the ability to bind RNA and single-stranded DNA using an OB fold formed by five antiparallel β-strands (45). Other members of the CSP family have previously been found to both stabilize RNAs and lead to antitermination (reviewed in reference 46). We used the *sgrS-S*-*rplL*T gene in the Δ*hfq* background to examine effects of other CSPs on the termination of SgrS. Six *csp* genes were each cloned in the pQE80L vector, as for CspD, and were ectopically expressed by the addition of IPTG. *cspE* and *cspG* were not able to be constructed in this vector. Northern blot analysis showed that overexpression of CspA, CspC, and CspI affected *sgrS-S-rplL*T transcription. However, these effects were different from that of CspD (Fig. S6). CspA and CspC produced a range of aberrant transcripts as Rof did (Fig. S6, lanes 3 and 5, denoted with an asterisk), suggesting that these Csps may affect Rho activity or Rho access to the transcribing sRNA. CspI overproduction increased both [T] and [RT], suggesting that it rather acts as a stabilizer for transcripts of *sgrS-S-rplL*T (Fig. S6, lane 8). CspB, CspF, and CspH did not significantly affect *sgrS-S-rplL*T transcription (Fig. S6, lanes 4, 6, and 7). Overall, these results suggested that CspD recognizes different RNAs and/or terminators than the other Csps. We think this could be one reason that no *csp* gene other than *cspD* was isolated by our screen.

10.1128/mbio.02371-22.6 FIG S6 Effects of overproduction of CSP proteins on the SgrS terminator. TM772 (Δ*hfq* Δ*sgrR-sgrS*) cells harboring the indicated plasmids were grown in LB-kanamycin, ampicillin medium. At an OD_600_ of 0.2, 0.2 mM IPTG was added to cultures to induce CSP proteins and incubation was continued for 60 min. Then, 0.4% arabinose was added to cultures to induce *sgrS-S*-*rplL*T and incubation was continued for 10 min. Total RNA was prepared and the RNA samples (5 μg) were subjected to Northern blotting using the SgrS-S probe. The asterisk indicates transcripts extending beyond the second terminator. Download FIG S6, PDF file, 0.2 MB. Copyright © 2022 Morita et al. 2022 Morita et al. https://creativecommons.org/licenses/by/4.0/ This content is distributed under the terms of the Creative Commons Attribution 4.0 International license.

### Global evaluation of CspD overproduction.

An intrinsic terminator is often used as a transcriptional termination signal for many E. coli genes, including within operons, leading to differential expression of genes in the operon (47). Because CspD overproduction interfered with these termination signals at the three sRNAs tested, CpsD was expected to affect termination globally. To obtain a global view of CspD function, we carried out an RNA-Seq analysis in the *hfq*^+^ background, comparing the vector control to pQE-CspD. Total RNAs were isolated from two independent cultures of each strain and analyzed using the RNA-Seq protocol described in Materials and Methods. A principal-component analysis (PCA) plot showed reproducibility of the two experiments (Fig. S7). Note that our RNA-Seq procedure was not optimized for capturing short RNAs such as the sRNAs. Because most of the sRNAs have no nearby downstream stop sites at the genome loci, the genome-wide effect on sRNA readthrough was not analyzed here.

10.1128/mbio.02371-22.7 FIG S7 Principal-component analysis (PCA) plot of CspD RNA-Seq data. TPM data in Table S1 were subjected to PCA. Download FIG S7, PDF file, 0.2 MB. Copyright © 2022 Morita et al. 2022 Morita et al. https://creativecommons.org/licenses/by/4.0/ This content is distributed under the terms of the Creative Commons Attribution 4.0 International license.

CspD overproduction altered the expression of 1,262 genes by more than 2-fold, of which expression of 655 genes increased and 607 genes decreased (Table S1). We focus here primarily on those that showed an increase. The increased expression of genes could result from a number of posttranscriptional effects, including stabilization of transcripts, decreased regulation by sRNAs, and increased readthrough from upstream genes.

10.1128/mbio.02371-22.9 TABLE S1 Effect of CspD overproduction on global gene expression. Columns A to D, gene information; columns E to H, the number of raw counts; columns I to L (TPM, transcripts per million kilobases); columns M to O, results of DESeq2 calculation. Genes that showed a >2-fold increase and an adjusted *P* value of <0.05 by CspD overproduction are highlighted in bold and pink. Genes that showed a >2-fold decrease and an adjusted *P* value of <0.05 by CspD overproduction are highlighted in bold and blue; columns P and Q, gene information; column R, information on operons, based on RegulonDB (45). The symbols > and < with dark colors indicate the leader position of an operon, whereas ≫ and ≪ with light colors indicate the second or latter positions on it. Column S, information on the genes in which internal termination sites were present, based on Adams et al. (41). [+] with gray indicates genes that contain one or more internal termination sites. Download Table S1, XLSX file, 0.7 MB. Copyright © 2022 Morita et al. 2022 Morita et al. https://creativecommons.org/licenses/by/4.0/ This content is distributed under the terms of the Creative Commons Attribution 4.0 International license.

**FIG 8:**
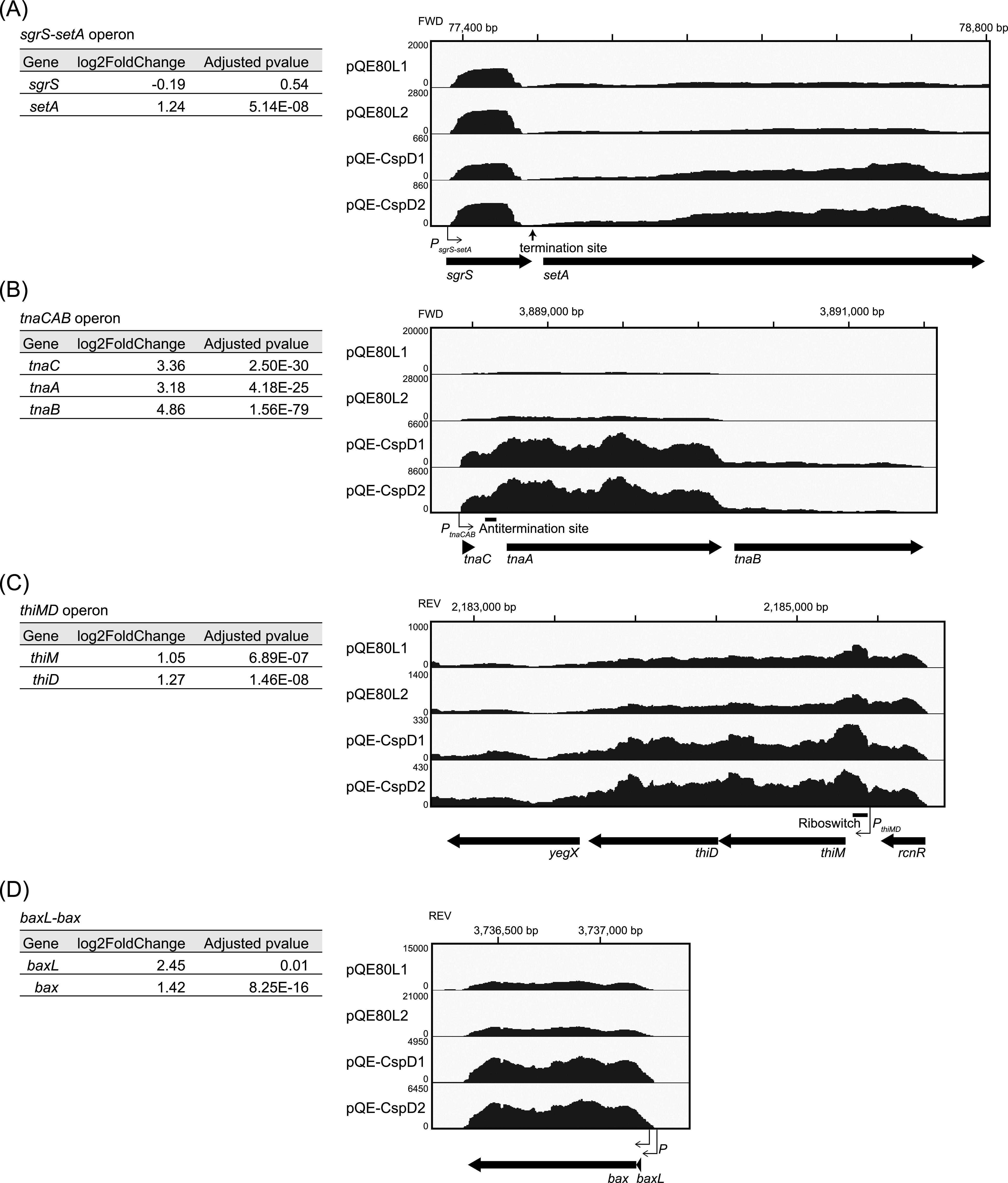
Selected operons affected by CspD overproduction. Fold change and Benjamini-Hochberg-adjusted *P* values for each gene (extracted from Table S1 in the supplemental material) are shown in the table to the left. Mapping of RNA-Seq expression data visualized by the Integrative Genomics Viewer (IGV) for the *sgrS-setA* operon (A), the *tnaCAB* operon (B), the *thiMD* operon (C), and *baxL-bax* (D) are shown. For a comparison on IGV, the vertical scale in each data set was determined based on the total number of mapped bases. The detailed method is described in Materials and Methods. Gene loci, promoters, and termination sites shown are based on information available at https://www.nichd.nih.gov/research/atNICHD/Investigators/storz/data-protocols/browsers#term-seq and the EcoCyc database (74).

We focused on operons with two or more genes, as defined by RegulonDB (48); operon organization is indicated in Table S1, column R. This allowed us to evaluate differential effects on reading through terminators between genes of operons as well as termination sites internal to genes (noted in Table S1, column S), which have been shown to promote premature termination in the early stage of transcription (42). Included in this set was the *sgrS* operon (Table S1, Fig. 8A), in which the downstream *setA* gene showed a significant increase, consistent with the increased expression of the *setA-lacZ* fusion used to identify CspD as an attenuation factor. Although the *sgrS* promoter was not fully activated under the tested condition and the RT products were less stable than the terminated SgrS due to the *hfq*^+^ background, the transcripts that extended across the SgrS terminator into *setA* increased in the CspD overproduction strain.

Other examples of operons affected by CspD overproduction were selected from the data in Table S1, and Integrative Genomics Viewer (IGV) images of the transcript pattern are shown in Fig. 8 and Fig. S8. The *tnaCAB* operon (Fig. 8B) encodes proteins involved in tryptophan catabolism; it is regulated by tryptophan-dependent transcriptional antitermination just downstream of *tnaC* (49). The dramatic increase for these genes is consistent with CspD overproduction overcoming the terminator in the leader. Two operons containing riboswitches, the *thiMD* operon and *gdx*, were also upregulated by CspD overproduction (Fig. 8C and Fig. S8A). The *thiMD* operon, encoding proteins involved in biosynthesis of thiamine pyrophosphate (TPP), is regulated by a TPP-sensing riboswitch at the upstream region of *thiM* (50); translation is inhibited when thiamine is abundant. Translation inhibition then leads to Rho-dependent mRNA degradation, via a region within the *thiM* ORF (51). *gdx* is regulated by a guanidine II riboswitch in its upstream region; translation is predicted to be turned up upon guanidine binding (52). Since these two riboswitches affect downstream translation in opposite directions, CspD may affect proper formation of RNA secondary structures rather than translation directly.

10.1128/mbio.02371-22.8 FIG S8 Additional examples of operons affected by CspD overproduction. Fold change and Benjamini-Hochberg-adjusted *P* values for each gene (extracted from Table S1) are shown in the table to the left. Mapping of RNA-Seq expression data visualized by IGV for *gdx* (A), the *mdtUJI* operon (B), the *yhi* operon (C), the *chiZPQ* operon (D), and the *thiCEFSGH* operon (E) is shown. For a comparison on IGV, the vertical scale in each dataset was determined based on the total number of mapped bases. The detailed method is described in Materials and Methods. Gene loci, promoters, and termination sites shown are based on information available at https://www.nichd.nih.gov/research/atNICHD/Investigators/storz/data-protocols/browsers#term-seq and the EcoCyc database. Download FIG S8, PDF file, 0.3 MB. Copyright © 2022 Morita et al. 2022 Morita et al. https://creativecommons.org/licenses/by/4.0/ This content is distributed under the terms of the Creative Commons Attribution 4.0 International license.

CspD also affected genes with known complex regulation at the 5′ end of mRNAs. For instance, a number of genes and operons containing small upstream ORFs with regulatory roles (53) were found to be increased upon CspD overproduction. This included *bax*, encoding a putative glycoside hydrolase (Fig. 8D), the *mdtUJI* operon, encoding spermidine export genes (Fig. S8B), and the operon associated with *yhiY* (Fig. S8C). These operons, as well as ones with sRNAs at the 5′ end (for instance, *chiZ* [Fig. S8D]) were shown to have transcription termination sites associated with them (Table S1, column S). In contrast, some of the operons that were regulated at the 5′ end were less affected by CspD overproduction. The expression profile of *thiC* and its following genes, encoding proteins involved in TPP biosynthesis, was similar in the CspD overproduction strain and in the vector control (Fig. S8E). The *thiC* operon is regulated by a TPP-sensing riboswitch, and its off state bound with TPP leads to the termination by Rho within *thiC* mRNA (51). These results suggest that overexpressing CspD does not modulate Rho activity.

### CspD functions under slow growth conditions with a poor carbon source.

**FIG 9:**
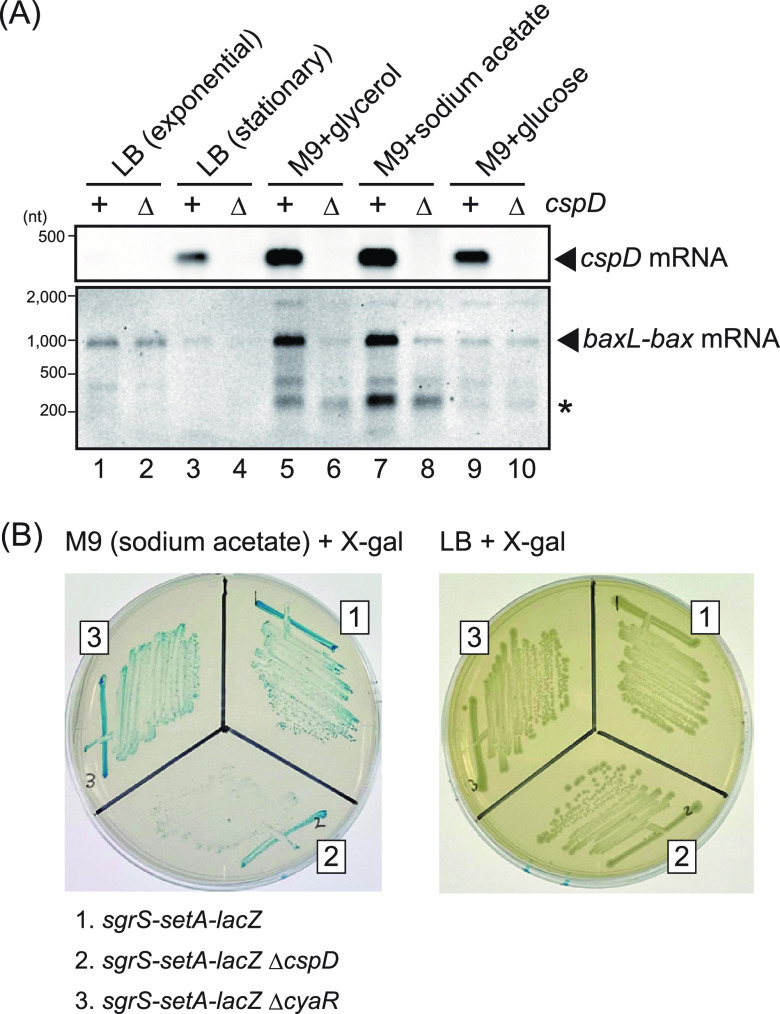
Endogenous CspD functions under slow growth. (A) Analyses of *cspD* expression and selected target mRNAs under several growth conditions. MG1655 and TM1043 (Δ*cspD*) cells were grown under the indicated conditions. In LB medium, cells were grown to an OD_600_ of 0.4 (exponential) or 2.0 (stationary). In M9 minimal medium supplemented with 0.1% glycerol, 10 mM sodium acetate, or 0.1% glucose, cells were grown to an OD_600_ of 0.2. RNA samples (1 μg) were subjected to Northern blotting using probes for *cspD* mRNA and *baxL-bax* mRNA. (B) Analysis of transcription readthrough at the SgrS terminator under slow growth conditions. TM1005 (*sgrS-setA-lacZ*), TM1173 (*sgrS-setA-lacZ* Δ*cspD*), and TM1045 (*sgrS-setA-lacZ* Δ*cyaR*) were grown on M9 sodium acetate (10 mM sodium acetate) plates or LB agar plates each containing 20 μg mL^−1^ X-Gal indicator. Incubation was continued for 3 days (M9 sodium acetate) or overnight (LB) at 37°C.

Since *cspD* was reported to be highly expressed under stationary phase and low growth rate conditions (28), the global functions of CspD, which were observed above, may play roles under these conditions. To address this, we compared the wild-type and Δ*cspD* strains under several culture conditions. The *cspD* mRNA was less expressed in wild-type cells grown to exponential phase in LB, and levels of target mRNA (*baxL-bax*) (Fig. 8D) were not affected by the Δ*cspD* mutant (Fig. 9A, lanes 1 and 2). By contrast, the *cspD* mRNA was highly expressed in other tested conditions and was most pronounced in M9 minimal medium with glycerol or sodium acetate as the carbon source (Fig. 9A, lanes 3 to 10). Under these two conditions, the full-length *baxL-bax* mRNA decreased in the Δ*cspD* mutant compared to wild-type cells (Fig. 9A, lanes 5 to 8). These results suggest that CspD may stabilize transcripts, as it does for sRNAs, and also may overcome RNA polymerase stalling during elongation. In addition, shorter transcripts were also detected under slow growth conditions both in the absence and presence of CspD (Fig. 9A, indicated with an asterisk). These bands are what might be expected for the upstream regulatory region.

We additionally tested the effect of the chromosomal *cspD* gene by using the *sgrS-setA-lacZ* allele. Cells were grown on M9 agar plates with sodium acetate as the carbon source and containing 5-bromo-4-choro-3-indolyl-β-d-galactopyranoside (X-Gal) as indicator. The *cspD*^+^ strain made blue colonies, while Δ*cspD* strains had reduced blue color (Fig. 9B, right panel, sectors 1 and 2), suggesting that transcription readthrough at the SgrS terminator occurred in a CspD-dependent manner. These two strains made similar colored colonies on LB agar plates containing X-Gal indicator (Fig. 9B, left panel, sectors 1 and 2). Together, these results indicate that CspD functions as an attenuator of transcription termination under slow growth conditions. Because the cAMP-cAMP receptor protein (CRP) complex regulates the expression of *cspD* (54) as well as *cyaR* (35, 55), we considered that CyaR might be involved in the CspD function. However, Δ*cyaR* strains harboring the *sgrS-setA-lacZ* allele made blue colonies comparable to the control strain (Fig. 9B, sector 3), indicating that CyaR is not responsible for the CspD function under these growth conditions.

## DISCUSSION

Intrinsic transcription terminators are characterized by a stem-loop followed by a run of U residues. For sRNAs, in which the terminator is a critical component of its ability to be bound by Hfq and therefore function for gene regulation, a poly(U) tail of seven or more residues is required. In general, poly(U) tails of the intrinsic terminator for mRNAs contain four to eight Us and are frequently disrupted by other nucleotides. Here, we searched for factors that modulated transcriptional termination to regulate sRNA signaling. Using a multicopy screen developed to evaluate readthrough at the SgrS terminator, we identified three protein factors, *cspD*, *ygjH*, and *rof*, and one sRNA, CyaR, as attenuation factors. The effects of the protein attenuators were also detected on the terminators of CyaR and GcvB. Consistent with our demonstration that readthrough products of SgrS-S do not function as an sRNA, overexpression of CspD and Rof, which both lead to a decrease in proper termination, reduced the regulation by SgrS of mRNA targets. Overall, these results identified the efficiency of intrinsic termination as an additional layer of regulation for global effects on sRNA signaling.

### Surprising effect of Rho on sRNA termination.

The terminators of sRNA genes are generally considered intrinsic or factor-independent terminators. Thus, it was surprising to identify Rof, a known inhibitor of the Rho termination factor (30), as an sRNA attenuator. We confirmed this effect was due to inactivation of Rho, as a point mutant of *rho* and treatment with the Rho inhibitor BCM had very similar effects to Rof overexpression (Fig. 5). This Rof effect was also observed at the terminator of sRNAs CyaR and GcvB. In addition, our screen isolated a DNA fragment containing *rhoL* (Fig. 1B). The *rhoL* gene is present at the leader region of the *rho* gene and is involved in the regulation of *rho* expression (56). An interpretation of these results is that Rho may contribute to sRNA production by contributing to appropriate termination to create mature sRNAs. A recent transcriptome analysis by Adams and colleagues proposed that Rho-dependent premature termination frequently happens, particularly in the early stage of transcription, such as within 5′-untranslated regions and internal to ORFs (42). However, little is currently known about how Rho causes termination of *sgrS-S* transcription; a *rut* site is likely not present within the *sgrS-S* region. To elucidate the involvement of Rho/Rof in sRNA production, further study will be needed. Disruption of Rho activity led to the appearance of a whole range of abnormal transcripts from the sRNA genes (Fig. 5), complicating the understanding of the role of Rho for these RNAs.

While the effects discussed here were found with Rof overexpression, deletion of *rof* did not show a significant phenotype (Fig. S4). The expression of the *yaeP-rof* operon is observed in slowly growing cells (30); future work to investigate the physiological role of Rof in sRNA signaling would be appropriate under such growth conditions.

### CspD disrupts termination and stabilizes sRNA transcripts.

CspD significantly increased readthrough of the three sRNAs examined, and it increased total transcripts from those genes (Fig. 2, Fig. 3). This increase in transcripts most likely reflected its ability to stabilize the readthrough transcript (Fig. 6). Our analyses suggest that the readthrough products of SgrS-S-*rplL*T are degraded by RNase E, whereas other RNase(s) are likely involved in the decay of properly terminated SgrS-S and SgrS itself. A previous study by gradient profiling by sequencing analysis found that CspD does not seem to associate with ribonucleases including RNase E *in vivo* (57). We propose that CspD binding to the nascent transcripts can protect them from decay by a variety of ribonucleases. At the same time, binding would seem to reduce proper termination within the tested sRNAs, strongly suggesting that binding happens cotranscriptionally. This would imply that one target of sRNA termination attenuation would be factors that prevent formation of an RNA stem-loop structure needed for intrinsic termination. Certainly, there is known regulation of RNA stem-loop structures in classical attenuation systems (49).

Consistent with the effect of CspD overproduction on specific sRNAs, we also saw broad changes in transcription across the genome. At least in some cases, this was consistent with readthrough of some transcription termination sites, again implicating cotranscriptional binding of CspD. In general, it is difficult to exactly determine a termination position *in vivo* due to rapid decay from the 3′ end of transcripts by exoribonucleases. However, some of the sites in which transcriptional readthrough was promoted by CspD are reported to be Rho dependent. We imagine that CspD, by binding to these transcripts, may regulate either utilization of *rut* sites or the efficiency of proper folding or recognition of regulatory sites, such as riboswitches or intrinsic terminators. Effects on genes with small ORF regulators are consistent with CspD overproduction disrupting the RNA structure, possibly masking sites needed for ribosome entry.

Of note, transcription elongation complexes can cause double-strand breaks of chromosomal DNA by colliding with replisomes, and thus transcriptional termination can maintain chromosome integrity by preventing their collision (58). Given an inhibitory effect of CspD on plasmid DNA replication *in vitro* (29), CspD might play a role in striking a balance between transcription and DNA replication.

That leaves unaddressed the specificity of Csp’s. In a previous study, three Csp’s (CspA, CspC, and CspE) were shown to have attenuator activities *in vivo* and *in vitro*, decreasing intrinsic termination at the internal terminator within the *metY-rpsO* operon (59). Increased expression of the downstream genes *nusA* and *rbfA* was observed when these Csp’s were overproduced. It is worth noting that no *csp* gene other than *cspD* was isolated by our screen and that the RNA-Seq analysis in the CspD overproduction strain did not show an increase in expression of *nusA* and *rbfA* previously seen with other Csp’s (Table S1). The pattern of readthrough at the SgrS-S-*rplL*T locus was very different for CspD overproduction, compared to CspA or CspC overproduction (Fig. S6). Therefore, CspD likely recognizes different mRNAs and/or terminators or acts differently once bound to these RNAs from the other Csp’s. Even within the two operons with TPP-responsive riboswitches, CspD had differential effects; while the *thiM* operon was up, the *thiC* operon was not significantly affected (Fig. 8C, Fig. S8E). Similarly, we found increased transcripts for multiple operons with small upstream ORFs (Fig. 8D and Fig. S8B and C), but certainly not for all such operons.

CspD uniquely has an extra acidic C-terminal tail not found in the other E. coli Csp proteins. Certainly, the expression patterns for the Csps also differ, with CspD highly expressed under low growth rate conditions and stationary phase, whereas CspA is expressed during cold shock; CspC and CspE are constitutively expressed (60). One can envision that Csps individually regulate a variety of terminator targets in response to the cognate condition under which they are activated.

In addition to the overproduction strain, we examined the effects of endogenous CspD under physiological conditions (Fig. 9). The effects of CspD were most striking during slow growth, possibly reflecting increased termination during elongation. Recent *in vivo* studies support the effects of different growth conditions on termination; in an earlier *in vitro* study, low UTP was found to increase termination efficiency (42, 47, 61). We imagine that CspD would function to overcome these obstacles. Additionally, the Fitness Browser database (62) indicates that a *cspD* mutant grows less effectively under some conditions, such as M9 minimal medium with α-ketoglutaric acid as a carbon source. Whether this phenotype or others reflects an effect on sRNA function remains to be determined, although such studies may best be done once we further understand whether redundancy exists for Csp regulation of sRNA termination.

### CyaR and YgjH remain to be understood.

CyaR, but not other class II sRNAs, was also identified in our screen. It seems likely that the activity of CyaR is indirect, via Hfq-dependent interaction with a target mRNA. Thus, a rim mutation in Hfq, expected to interfere with CyaR pairing but not with its accumulation, abolished the effect on the SgrS reporter; point mutations in one of the seed pairing regions of CyaR also abolished this activity. This is an intriguing finding, suggesting that under conditions of CyaR expression, this antitermination activity could serve as a feedback mechanism, dampening levels of other sRNAs that may compete for Hfq and possibly dampening levels of CyaR itself. This possibility remains to be further explored.

YgjH was the weakest of the factors identified here; it had a modest effect on terminator readthrough for all three tested sRNAs. It has significant homology to a tRNA binding protein (31), suggesting it may bind RNAs, but very little is currently known about it. Ribosome profiling suggests very low levels of the protein in morpholinepropanesulfonic acid-glucose medium and increased expression in rich medium (63).

### Summary.

This study proposes that sRNA signaling could become a model for studying the modulation and efficiency of intrinsic termination. Recent RNA-Seq analyses have revealed that the modulation of intrinsic termination and readthrough is more widespread than previously thought (47, 64). However, the mechanisms by which the termination is regulated remain unexplored. The genetic screen used for our study also yielded clones making white colonies, possibly resulting from enhanced intrinsic termination of SgrS. Our findings here provide a system for defining the nature of RNAs recognized by CspD and how it might differ from other Csp’s. Further studies and screening methods with other sRNA terminators and/or reporter genes are needed to fully understand modulation of intrinsic termination.

## MATERIALS AND METHODS

### Growth conditions.

Cells carrying the indicated plasmids were grown at 37°C in LB medium or M9 minimal medium (without Casamino Acids) supplemented with ampicillin (50 μg mL^−1^), zeocin (25 μg mL^−1^), and kanamycin (15 μg mL^−1^) when necessary. Concentrations of the carbon sources added in the medium are described in the figure legends. Overnight cultures were diluted 100-fold unless otherwise specified into the same fresh medium, unless indicated otherwise. Cell growth was monitored by determining the OD_600_.

### Bacterial strains and plasmids.

E. coli K-12 strains and plasmids used in this study are listed in Table S2 in the supplemental material. MG1655 was used as the parent strain in the multicopy screen and RNA-Seq analysis; in other experiments, IT1658 (W3110*mlc*) was used as the wild-type strain. Oligonucleotides and gBlocks (IDT) used for strain and plasmid construction are listed in Table S2. The *Cp17-sgrS-setA-lacZ* and *Cp17-sgrS*Δ*T-setA-lacZ* alleles were constructed using λ Red-mediated recombineering with gBlock gene fragments g1 and g2 in strain NM580, which contains *P_BAD_*-*ccdB* upstream of *lacZ* at the chromosomal *lac* locus (65).These alleles as well as mutations used in this study were moved into new backgrounds by P1 transduction, and where indicated, marker genes were removed using plasmid pCP20 (66).

10.1128/mbio.02371-22.10 TABLE S2 Strains and plasmids used in this study, and DNA oligos and gBlocks used in this study. Download Table S2, XLSX file, 0.01 MB. Copyright © 2022 Morita et al. 2022 Morita et al. https://creativecommons.org/licenses/by/4.0/ This content is distributed under the terms of the Creative Commons Attribution 4.0 International license.

Plasmids pTWV-CspD, pTWV-YaeP-Rof, and pTWV-YgjH were constructed as follows: one of the original plasmids from screening experiments was used to amplify the DNA fragment containing *cspD*, *yaeP-rof*, and *ygjH* with primers 2136/2121, 2124/2123, and 2125/2126, respectively. The amplified DNA fragments were digested with HindIII and EcoRI and then cloned into pTWV228. Plasmids pQE-CspD, pQE-YgjH, pQE-Rof, and pQE-YaeP were constructed as follows: one of the original plasmids was used to amplify the DNA fragment containing *cspD*, *ygjH*, *rof*, and *yaeP* with primers 2051/2052, 2106/2107, 2108/2109, and 2110/2111, respectively. The amplified DNA fragments were cloned into pQE80L by *in vitro* recombination using the In-Fusion HD cloning kit (TaKaRa Bio USA). The plasmid pQE-CspD-FLAG was constructed as follows: gBlock gene fragment g74 was used to amplify the DNA fragment containing *cspD-FLAG* with primers 2152/2224. The amplified DNA fragment was digested with EcoRI and HindIII and then cloned into pQE80L. Plasmids pCyaR-*rplL*T and pGcvB-*rplL*T were constructed to digest the gBlock gene fragments g67 and g73, respectively, with XbaI and HindIII and then cloned into pAraX. Plasmids in the pQE-Csp series were constructed as follows: chromosomal DNA of MG1655 was used to amplify the DNA fragment containing *csp* genes with primers 2251/2252 (*cspA*), 2253/2254 (*cspB*), 2255/2256 (*cspC*), 2259/2260 (*cspF*), 2263/2264 (*cspH*), and 2265/2266 (*cspI*). The amplified DNA fragments were digested with EcoRI and HindIII and then cloned into pQE80L.

### β-Galactosidase assays.

Colonies from a lactose MacConkey (Amp) plate were transferred into 1 mL of fresh chilled LB medium and suspended with a sterile micropipette tip. Cell density (OD_600_) was determined by using a part of the suspension. The remaining suspension was used for the β-galactosidase assay described by Miller (67).

### Northern blotting.

Total RNAs were isolated as described elsewhere (68). To detect SgrS-S, SgrS, CyaR, GcvB, and 5S rRNA, RNA samples were resolved by 10% polyacrylamide gel electrophoresis in the presence of 7 M urea (Invitrogen). To detect *mdtJI* mRNA, *ptsG* mRNA, *yigL* mRNA, and tmRNA, RNA samples were resolved by 1% agarose gel electrophoresis in the presence of formaldehyde. To detect *cspD* mRNA and *baxL-bax* mRNA, RNA samples were resolved by 1.5% agarose gel electrophoresis in the presence of formaldehyde. RNAs in the gel were blotted onto a nylon membrane that was positively charged (Roche). The RNAs were visualized by using a detection system with digoxigenin (DIG; Roche) and then captured and quantified using the imaging system ChimiDoc XRS Plus (Bio-Rad). The following RNA probes were prepared by the DIG RNA labeling kit (Roche): the antisense corresponding to the +168 to +198 portion of *sgrS* (SgrS-S probe); the antisense corresponding to the +1 to +35 portion of *cyaR* (CyaR probe); the antisense corresponding to the +1 to +40 portion of *gcvB* (GcvB probe); the antisense corresponding to the +33 to +72 portion of 5s rRNA (5S rRNA probe); the antisense corresponding to the −40 to +160 region relative to the AUG start codon of *mdtJ* (*mdtJ* probe); the antisense corresponding to the −48 to +152 region relative to the AUG start codon of *ptsG* (*ptsG* probe); the antisense corresponding to the +1 to +200 region relative to the AUG start codon of *yigL* (*yigL* probe); the antisense corresponding to the −100 to +100 region relative to the AUG start codon of *cspD* (*cspD* probe); the antisense corresponding to the −63 to +137 region relative to the AUG start codon of *bax* (*baxL-bax* probe). The tmRNA probe of a 363-bp DNA fragment was prepared by PCR using DIG-dUTP.

### Western blotting.

Cells were harvested from cultures (500 μL) by centrifugation, and the cell pellets were suspended with NuPAGE LDS sample buffer. The sample was resolved on 4% to 12% bis-Tris NuPAGE protein gel (Invitrogen) in morpholineethanesulfonic acid-SDS running buffer (Invitrogen), and then transferred onto a polyvinylidene difluoride membrane using the iBlot 2 dry blotting system (Thermo Fisher Scientific). The membranes were treated with an anti-FLAG monoclonal antibody (Sigma-Aldrich) and anti-GroEL polyclonal antibodies (Merck). Signals were visualized with the Lumi-Light Western blotting substrate (Merck) and then captured using the imaging system ChimiDoc XRS Plus (Bio-Rad).

### Pulldown assay.

Cells were grown in 80 mL of LB medium at 37°C. At an OD_600_ of 0.2, 0.2 mM IPTG was added and incubation was continued for 1 h. Then, 0.4% arabinose was added, and incubation was continued for 10 min. Cells were harvested and washed with 1 mL immunoprecipitation (IP) buffer (20 mM Tris-HCl [pH 8.0], 0.1 M KCl, 5 mM MgCl_2_, 10% glycerol, and 0.1% Tween 20). The cell pellet was suspended in 1 mL IP buffer. The cell suspension was crushed with a μT-01 beads crusher (Titec) with ~0.350- to 0.500-mm glass beads. After centrifugation at 10,000 × *g* for 10 min at 4°C, the supernatant (crude extract) was incubated with 40 μL of anti-FLAG M2-agarose suspension (Sigma-Aldrich) in 1 mL of IP buffer for 25 min at 4°C. The agarose was collected by centrifugation at 10,000 × *g* for 1 min at 4°C and was washed three times with 1 mL of IP buffer. The proteins bound to the beads were eluted with 40 μL of IP buffer containing 0.4 mg mL^−1^ FLAG peptide (Sigma-Aldrich) and used as the bound fraction. To analyze proteins, crude extract (0.4 μL) and the bound fraction (0.4 μL) were subjected to Western blotting. To analyze RNAs, crude extract (2 μL or 0.1 μL) and bound fraction (2 μL or 0.1 μL) for SgrS-S or tmRNA, respectively, were treated with phenol, precipitated, and washed with ethanol. Each precipitant was dissolved in RNA buffer. The RNA samples were subjected to Northern blotting.

### Library preparation and RNA sequencing.

MG1655 cells harboring pQE80L or pQE-CspD were grown in LB medium. At an OD_600_ of 0.2, 1 mM IPTG was added to cultures and incubation was continued for 30 min. Total RNAs were isolated from two independent cultures of each strain, as described elsewhere (68), then purified by RNeasy mini spin column (Qiagen). Ten micrograms of RNA sample was subjected to rRNA removal using the Ribominus transcriptome isolation kit (yeast and bacteria; Invitrogen). Libraries were constructed using NEBNext Ultra II directional RNA library prep kit for Illumina (New England Biolabs). The library molar concentration and quality were measured by TapeStation using high-sensitivity D1000 screen tape (Agilent). The libraries were sequenced using HiSeq XTen by BGI (Hong Kong). The Illumina paired-end sequencing data were preprocessed with fastp (Q15) (69), performing adapter trimming and quality filtering. The reads were mapped to the Escherichia coli strain K-12 substrain MG1655 reference genome (RefSeq accession number NC_000913.3) with Bowtie2 version 2.3.5.1 (70). Read counts per gene were calculated with HTseq 0.11.2 (71). Relative read counts for each gene were provided as transcripts per million (TPM). The TPM data were subjected to PCA. Fold changes, *P* values, and Benjamini-Hochberg-adjusted *P* values for a differential expression analysis were calculated by using DESeq2 version 1.30.1. In calculations for TPM or DESeq2, the read counts for rRNA and tRNA genes were subtracted from the total read counts. Information on genes, operons, and the termination sites internal to genes were obtained from RegulonDB 10.9 (48) and Adams et al. (42), respectively. Integrative Genomics Viewer (IGV_2.8.4) (72) was used to visualize transcriptome profiles. For a comparison on IGV, the vertical scale for each data set was determined based on the total number of mapped bases, as follows: 20,167,065,437 (pQE80L1), 28,173,790,850 (pQE80L2), 6,684,884,557 (pQE-CspD1), and 8,626,012,370 (pQE-CspD2).

### Data availability.

RNA-Seq data reported in this study have been deposited in the DDBJ database under the accession number PRJDB13439.
