# Supplementary figures and images for: Identification of Attenuators of Transcriptional Termination: Implications for RNA Regulation in Escherichia coli
Source: mBio. 2022 Oct 13;13(6):e02371-22. doi: 10.1128/mbio.02371-22 (PMC9765468; doi:10.1128/mbio.02371-22)

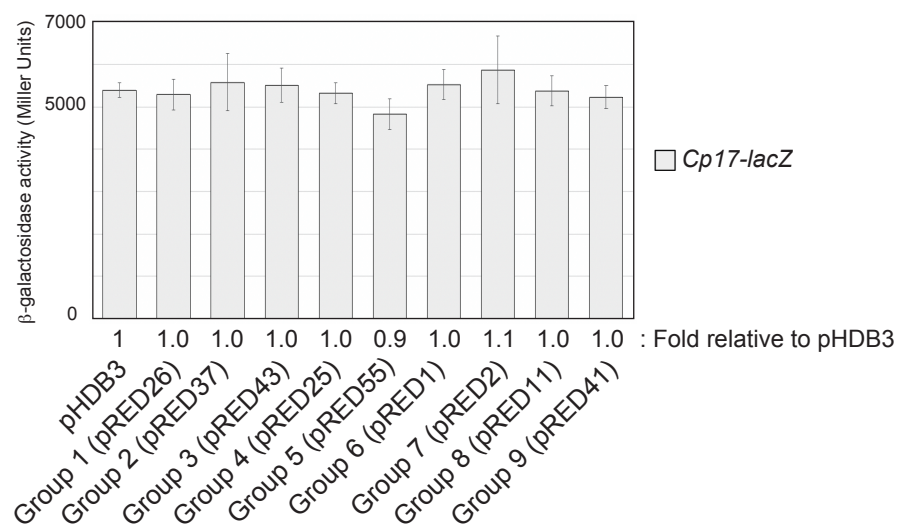

Supplemental Fig. S1, Morita, *et al*

Supplement: FIG S1 [file mbio.02371-22-s0001.pdf]

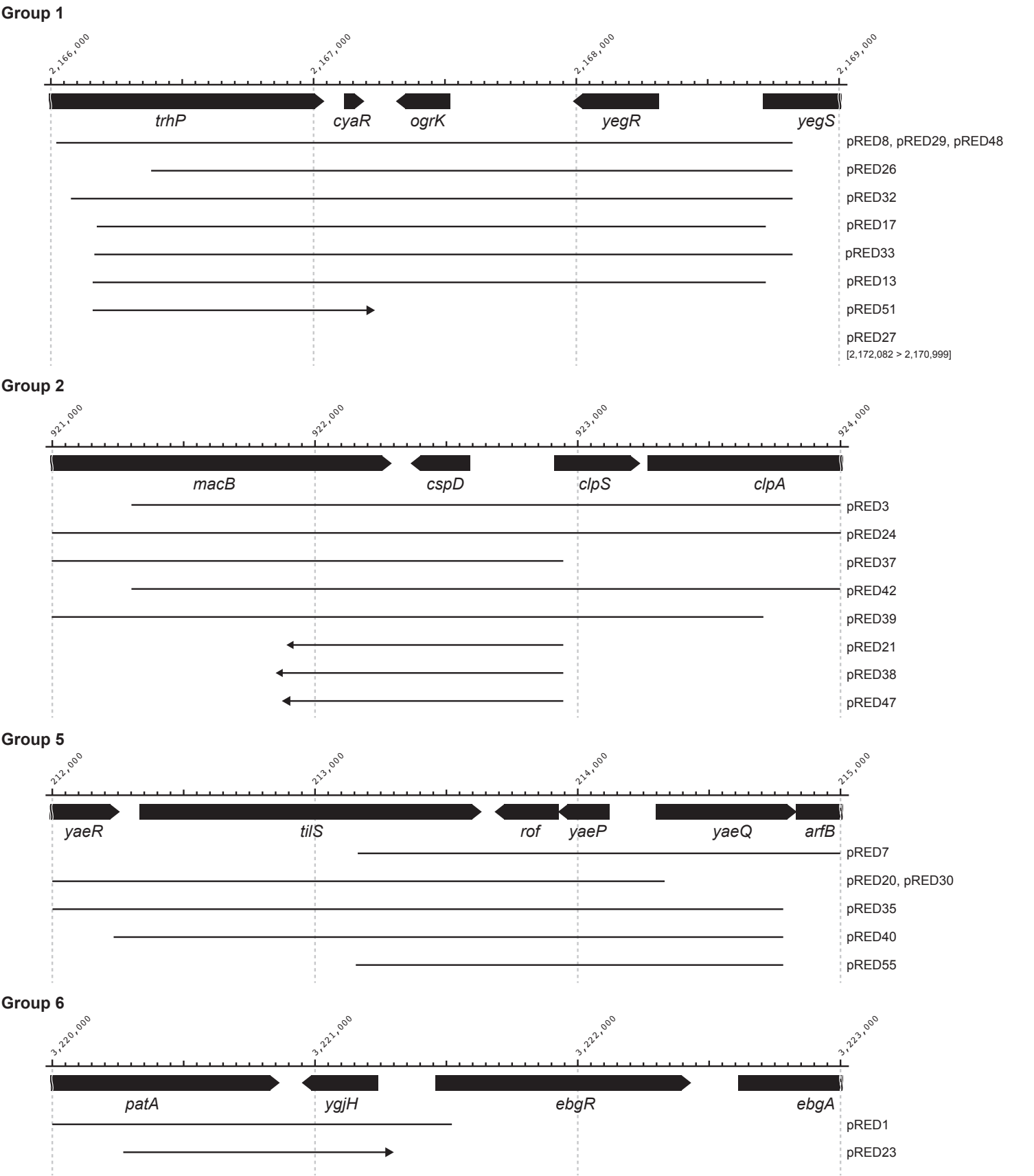

Supplemental Fig. S2, Morita, *et al*

Supplement: FIG S2 [file mbio.02371-22-s0002.pdf]

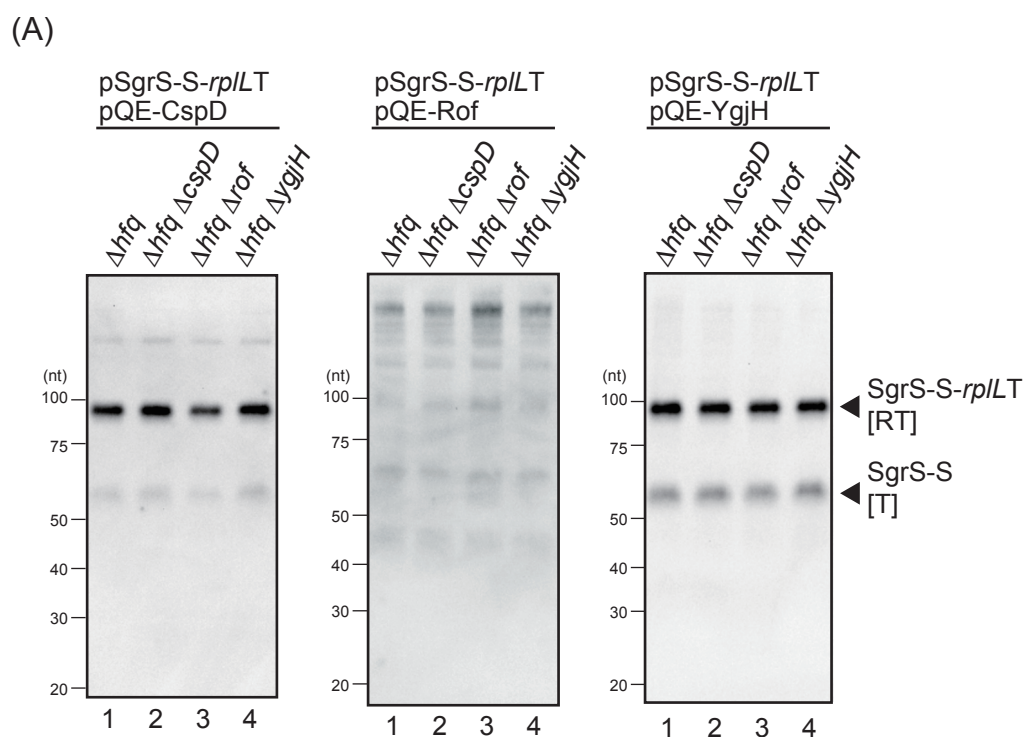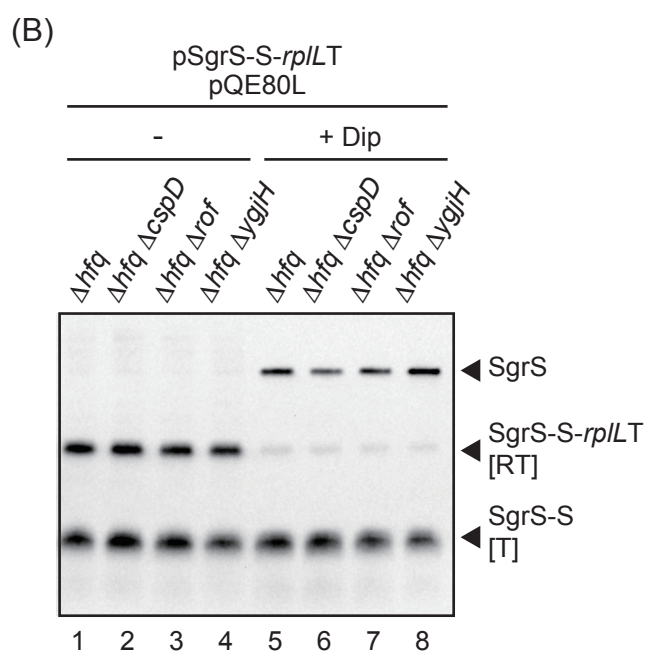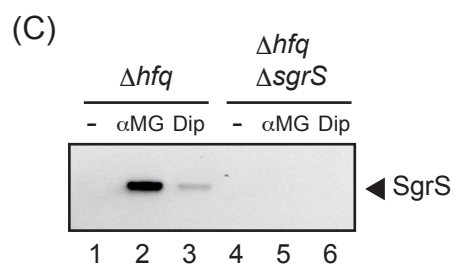

Supplemental Fig. S4, Morita, *et al*

Supplement: FIG S4 [file mbio.02371-22-s0004.pdf]

(A)

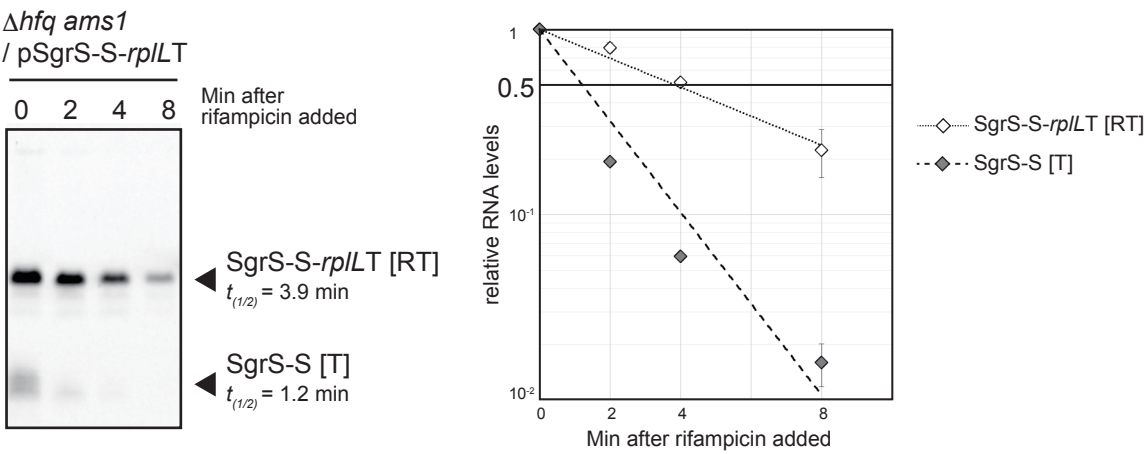

(B)

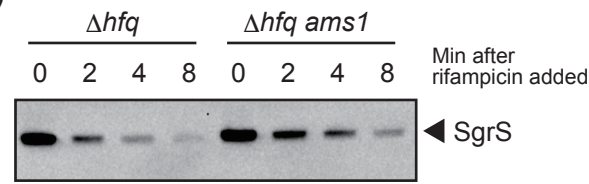

Supplemental Fig. S5. Morita, *et al*

Supplement: FIG S5 [file mbio.02371-22-s0005.pdf]

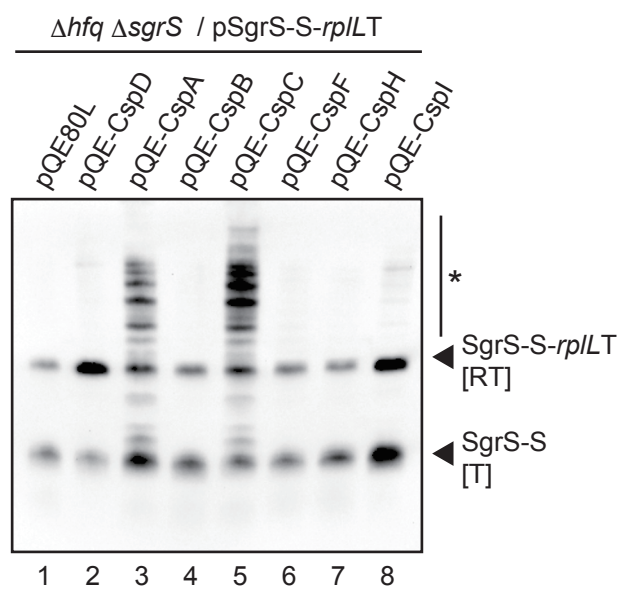

Supplemental Fig. S6, Morita, *et al*

Supplement: FIG S6 [file mbio.02371-22-s0006.pdf]

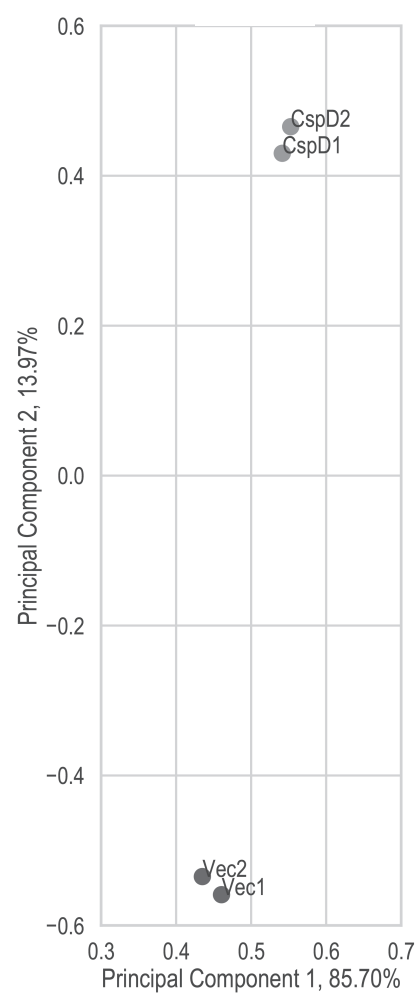

Supplemental Fig. S7, Morita, *et al*

Supplement: FIG S7 [file mbio.02371-22-s0007.pdf]
