# Supplementary material for: Identification of Attenuators of Transcriptional Termination: Implications for RNA Regulation in Escherichia coli
Source: mBio. 2022 Oct 13;13(6):e02371-22. doi: 10.1128/mbio.02371-22 (PMC9765468; doi:10.1128/mbio.02371-22)
Supplement: FIG S3 [file mbio.02371-22-s0003.pdf]

(A)

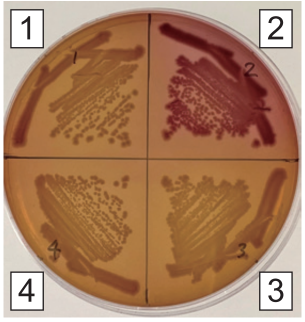

TM1013 (*sgrS-setA-lacZ* Δ*sgrS*)
1. pBR-lac
2. pCyaR
3. pMcaS
4. pChiX

(B)

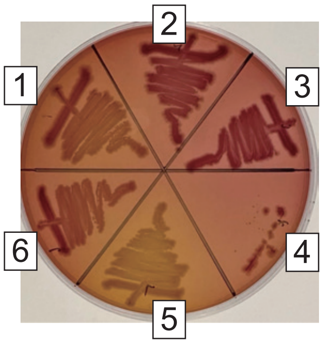

TM1013 (*sgrS-setA-lacZ* Δ*sgrS*)
1. pTWV228
2. pTWV-CspD (native promoter)
3. pTWV-YaeP-Rof (native promoter)
4. pQE-Rof (IPTG(lactose)-inducible promoter)
5. pQE-YaeP (IPTG(lactose)-inducible promoter)
6. pTWV-YgjH (native promoter)

(C)

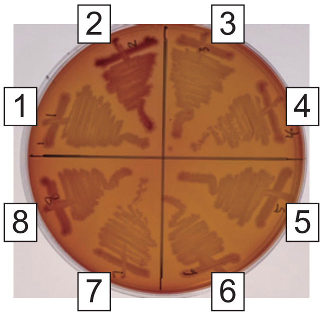

1. TM1013 (*sgrS-setA-lacZ* Δ*sgrS*) / pBR-lac
2. TM1013 / pCyaR
3. TM1030 (TM1013 Δ*hfq*) / pBR-lac
4. TM1030 / pCyaR
5. TM1037 (*hfqR16A sgrS-setA-lacZ*) / pBR-lac
6. TM1037 / pCyaR
7. TM1038 (*hfqY25D sgrS-setA-lacZ*) / pBR-lac
8. TM1038 / pCyaR

(D)

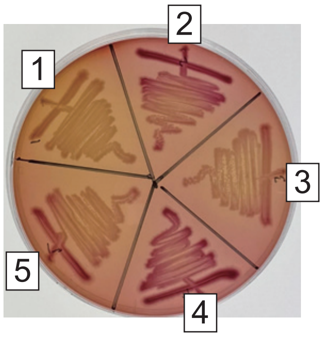

1. TM1013 (*sgrS-setA-lacZ* Δ*sgrS*) / pBR-lac
2. TM1013 / pCyaR
3. TM1013 / pCyaR A44T
4. TM1013 / pCyaR T47A
5. TM1013 / pCyaR G38C, G39A, A40T

```
                 CAU    U  A
                  ↑     ↑  ↑
CyaR  UGUACCAGGAACCACCUCCU
      31                 50
```

(E)

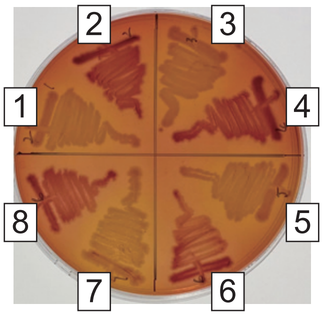

1. TM1005 (*sgrS-setA-lacZ*) / pBR-lac
2. TM1005 / pCyaR
3. TM1073 (TM1005 Δ*cspD*) / pBR-lac
4. TM1073 / pCyaR
5. TM1074 (TM1005 Δ*ygjH*) / pBR-lac
6. TM1074 / pCyaR
7. TM1075 (TM1005 Δ*rof*) / pBR-lac
8. TM1075 / pCyaR

Supplemental Fig. S3, Morita, *et al*
