# Supplementary material for: Identification of Attenuators of Transcriptional Termination: Implications for RNA Regulation in Escherichia coli
Source: mBio. 2022 Oct 13;13(6):e02371-22. doi: 10.1128/mbio.02371-22 (PMC9765468; doi:10.1128/mbio.02371-22)
Supplement: FIG S8 [file mbio.02371-22-s0008.pdf]

(A) *gdx*

Gene	log2FoldChange	Adjusted pvalue
gdx	2.51	4.29E-18

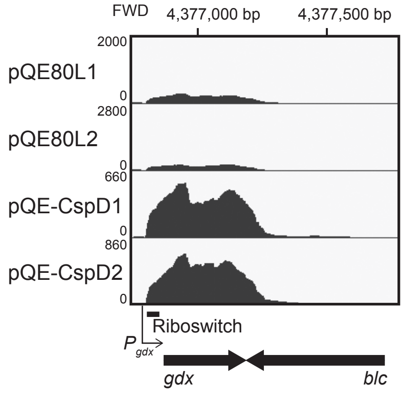

(B) *mdtUJI* operon

Gene	log2FoldChange	Adjusted pvalue
mdtU (*ydgV*)	1.96	2.71E-09
mdtJ	2.92	6.62E-18
mdtI	2.78	1.12E-12

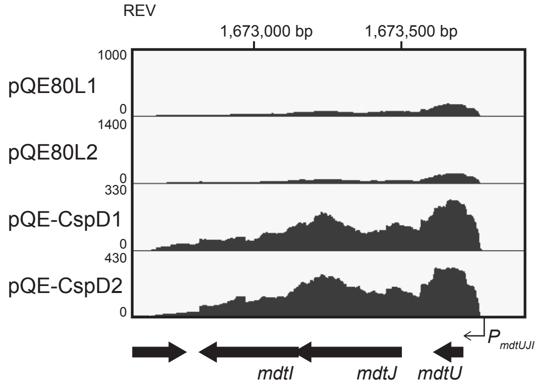

(C) *yhi* operon

Gene	log2FoldChange	Adjusted pvalue
yhiY	1.30	1.08E-06
yhiI	1.40	2.85E-10
rbbA	0.87	0.002
yhhJ	0.92	0.0004

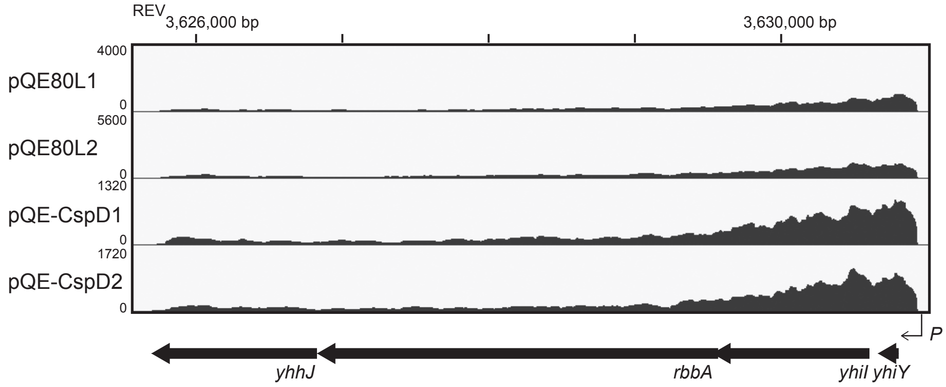

(D) *chiZPQ* operon

Gene	log2FoldChange	Adjusted pvalue
chiZ	ND	ND
chiP	1.09	1.02E-07
chiQ	1.94	1.58E-06

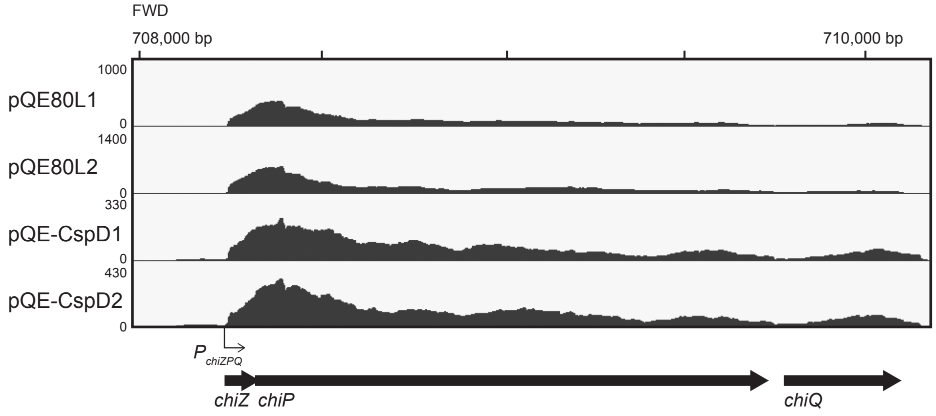

(E) *thiCEFSGH* operon

Gene	log2FoldChange	Adjusted pvalue
thiC	-0.26	0.34
thiE	-0.06	0.90
thiF	-0.09	0.86
thiS	0.63	0.78
thiG	-0.07	0.89
thiH	0.35	0.34

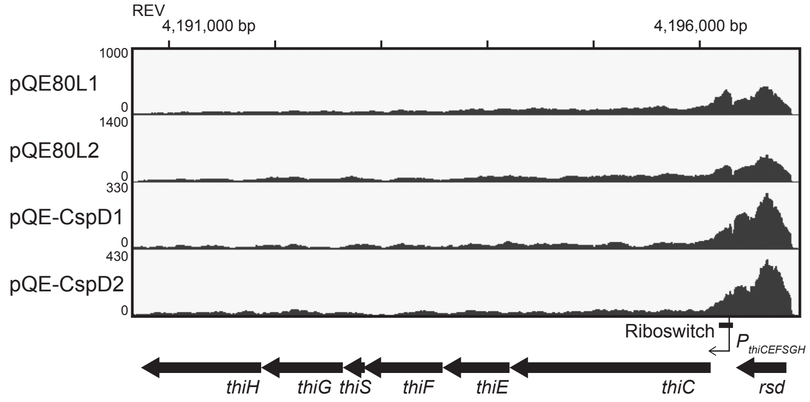

Supplemental Fig. S8, Morita, *et al*
